# Effects of Biogas Slurry, Biochar, and Mineral Fertilizer Co-Application on Net Ecosystem Carbon Balance and Ecosystem Service Value in Greenhouse Farmland

**DOI:** 10.3390/plants15132087

**Published:** 2026-07-04

**Authors:** Qinglin Sa, Jian Zheng, Yan Wang, Xuqin Fu, Shikun Sun, Yongde Gan

**Affiliations:** 1 College of Energy and Power Engineering, Lanzhou University of Technology, Lanzhou 730050, China; saql20271417@163.com; 2 School of Civil and Hydraulic Engineering, Lanzhou University of Technology, Lanzhou 730050, China; saqinglin0902@163.com; 3 Key Laboratory of Multi-Supply System with Solar Energy and Biomass, Lanzhou 730050, China; 4 School of Green Energy and Storage, Lanzhou University of Technology, Lanzhou 730050, China; 221080791001@lut.edu.cn; 5 College of Water Resources and Architectural Engineering, Northwest A&F University, Yangling 712100, China; sksun@nwafu.edu.cn; 6 School of Civil Engineering and Water Resources, Qinghai University, Xining 810016, China; saql20271417@yeah.net

**Keywords:** biogas slurry, biochar, economic benefit, net ecosystem carbon balance, ecosystem services, multi-objective evaluation model

## Abstract

In intensive greenhouse agriculture, irrational fertilization practices can exacerbate carbon emissions and impair ecosystem service functions. To address this issue, biogas slurry and biochar were introduced as waste-derived substitutes for mineral fertilizer, and the effects of different fertilization strategies on the net ecosystem carbon balance (*N*_ECB_) and ecosystem service value (ESV) of greenhouse tomato (*Solanum lycopersicum* L.) production systems over two growing seasons (spring–summer and autumn–winter) were systematically evaluated. When economic return was prioritized, the treatment with 25% biogas slurry substituting for mineral fertilizer (BS25) performed best, with ESVs of 641,606.83 and 629,987.37 CNY ha^−1^ in the spring–summer and autumn–winter seasons, respectively; the treatment with 50% biogas slurry substitution (BS50) ranked second, and both treatments were significantly superior to the others (*p* < 0.05). When the objective was to enhance carbon sink capacity while maintaining high yield, the treatment with 75% biogas slurry combined with biochar substituting for mineral fertilizer (BS75 + C) showed the best overall performance, with *N*_ECB_ values of 6.30 and 6.34 t ha^−1^ in the two respective seasons, while also demonstrating clear advantages in soil organic matter accumulation and atmospheric regulation. Based on the VIKOR model with AHP-CRITIC combined weighting, BS75 + C was identified as the optimal option. However, the most suitable fertilization strategy depends on management objectives: BS25 is recommended when maximizing short-term economic return is the primary goal, whereas BS75 + C is preferable for enhancing carbon sink capacity and ecological benefits. Considering both ecosystem service value and comprehensive performance, BS50 and BS75 + C are recommended as sustainable fertilization strategies for greenhouse tomato production.

## 1. Introduction

In the context of global climate change, achieving both agricultural greenhouse gas mitigation and food security has become a key issue in sustainable agricultural development. Agriculture is one of the major anthropogenic sources of greenhouse gas emissions. However, the contribution of agriculture to global greenhouse gas emissions varies considerably among different estimation approaches and emission categories. Previous studies have shown that agriculture is a major source of CO_2_, CH_4_, and N_2_O emissions, with agricultural activities accounting for approximately two-thirds of global anthropogenic N_2_O emissions [1,2]. Therefore, promoting carbon sequestration and emission reduction in agricultural systems is an important pathway toward achieving the dual goals of climate security and food security. Compared with open-field systems, greenhouse agriculture is characterized by higher energy consumption, more concentrated material inputs, and more intensive human management, all of which make its carbon cycling processes more complex. Moreover, greenhouse systems typically receive substantially greater fertilizer and irrigation inputs than open-field systems, resulting in higher greenhouse gas emission intensities, altered carbon turnover processes, and different ecosystem service compositions. Consequently, greenhouse agriculture exhibits not only significant carbon emission characteristics but also a certain potential for carbon sequestration. Accordingly, optimizing soil management practices to enhance carbon sequestration capacity and reduce net carbon emissions while maintaining high and stable vegetable yields has become an urgent issue for the environmental sustainability of protected agriculture.

In intensive greenhouse production, high mineral fertilizer input is a common management practice for sustaining high crop yields. However, long-term excessive fertilization often fails to continuously increase yield and instead leads to reduced fertilizer use efficiency, soil quality degradation, and increased environmental risks [3,4]. In this context, alternative fertilization strategies based on the resource utilization of agricultural wastes have attracted widespread attention. Biogas slurry is rich in readily available nutrients and has considerable potential to substitute for mineral fertilizers, but its nutrient forms are highly active and therefore prone to loss. In addition, excessive or improper application of biogas slurry may increase environmental risks, including NH_3_ volatilization, N_2_O emissions, nitrate leaching, soil salinization, pathogen transmission, and the accumulation of potentially toxic elements. By contrast, biochar possesses an abundant pore structure, a large specific surface area, and strong adsorption capacity, enabling it to retain and slowly release nutrients, thereby improving the soil environment and enhancing nutrient use efficiency [5,6]. Previous studies have demonstrated that the application of biogas slurry can stimulate soil carbon and nitrogen transformations, thereby enhancing crop yield [7]. However, once large amounts of readily decomposable organic carbon and ammonium nitrogen in biogas slurry enter the soil, soil respiration and nitrogen transformation processes may also be stimulated, thereby increasing the risks of NH_3_ volatilization and CO_2_ and N_2_O emissions [8]. Compared with biogas slurry, biochar, as a relatively stable carbon-based material, can not only improve soil structure and nutrient and water retention capacity, but also increase crop yield, quality, and biomass [9], while exerting regulatory effects on greenhouse gas emissions [10]. Nevertheless, the effects of biochar on crop productivity, soil fertility, carbon sequestration, and greenhouse gas emissions remain a matter of scientific debate. Previous studies have reported positive, neutral, and even contrasting responses depending on feedstock type, pyrolysis conditions, application rate, soil properties, climatic conditions, and cropping systems [11]. For example, Iqbal et al. [12] reported that biochar applied at 50 t ha^−1^ produced a pronounced mitigation effect on greenhouse gas emissions. Similarly, Yin et al. [13] found that biochar reduced CH_4_ and CO_2_ emissions from paddy fields while increasing rice yield. Haider et al. [14] further suggested that biochar may regulate greenhouse gas emissions through its effects on soil microbial processes and nutrient cycling, although its impacts on N_2_O and CH_4_ remain uncertain. Overall, biogas slurry and biochar have respective advantages in nutrient supply and soil improvement, and they may exert complementary effects in nutrient retention and slow-release regulation. However, existing studies have mainly focused on the effects of single-material application, and the application potential of combining biogas slurry and biochar in intensive greenhouse farmland remains insufficiently understood. Moreover, limited information is available regarding how different biogas slurry substitution ratios combined with biochar influence carbon balance, crop productivity, and ecosystem services simultaneously in intensive greenhouse systems. Under high-input greenhouse conditions, the combined effects of different biogas slurry substitution ratios and different co-application patterns with biochar and mineral fertilizers on farmland net carbon balance, crop yield, and soil fertility improvement have not yet been systematically evaluated. Therefore, such studies are of great significance for optimizing greenhouse nutrient management and achieving synergies between stable production and emission reduction.

Agricultural management practices not only affect farmland’s carbon dynamics and crop production, but also exert important influences on the overall functioning of farmland ecosystems. It is therefore necessary to evaluate their effects from a more comprehensive perspective. Ecosystem service value (ESV) is an important approach for monetarily characterizing the products and services provided by ecosystems to humans. It encompasses provisioning services, regulating services, supporting services, and cultural services, and serves as an important tool for evaluating the comprehensive benefits of farmland ecosystems [15,16]. Previous studies have demonstrated that farmland management practices can markedly influence both ecological and economic outcomes. For example, Schipanski et al. [17] showed that cover crops significantly improved the ecological benefits of farmland in Pennsylvania, although their contribution to direct economic returns was limited. Sandhu et al. [18] reported that organic farming in Canterbury, New Zealand, outperformed conventional farming in both ecological and economic terms. Similarly, Hu et al. [19] found that a specific drip irrigation method combined with lignite-based carbon organic fertilizer enhanced both productivity and ecosystem service value in saline–alkali farmland. These findings indicate that evaluating agricultural management practices solely from a single dimension, such as yield or greenhouse gas emissions, is insufficient to fully reflect their overall effects. Therefore, it is necessary to introduce the ESV evaluation framework into greenhouse farmland management research to comprehensively assess the ecological and productive value of the synergistic “biogas slurry–biochar–mineral fertilizer” management model. On this basis, the present study further combines the ecosystem service value evaluation framework with the combination-weighting VIKOR model to evaluate the comprehensive effects of different management scenarios, thereby providing a new analytical perspective for the multi-objective optimization of greenhouse farmland management practices.

Against this background, the present study focuses on an intensive greenhouse tomato cultivation system. Under equal nutrient inputs and identical irrigation conditions, treatments with different substitution ratios of biogas slurry for mineral fertilizers, with or without biochar application, were established to systematically evaluate the effects of this synergistic management model on farmland net carbon balance, greenhouse gas emissions, crop yield, and ecosystem service value. Specifically, this study aims to elucidate the responses of the ecological and productive functions of greenhouse farmland to different co-application ratios and to identify an optimized management mode that balances carbon sequestration and emission reduction, yield stability and improvement, and ecological benefits. We hypothesize that a moderate substitution of mineral fertilizer with biogas slurry can help maintain relatively high crop yields and thereby enhance the supply value of agricultural products, whereas a higher proportion of biogas slurry combined with biochar may increase regulating service value by enhancing carbon sequestration, reducing greenhouse gas emissions, and promoting soil organic matter accumulation. Therefore, trade-offs are expected among economic return, crop yield, *N*_ECB_, carbon sink capacity, and ecosystem service value under different fertilization strategies. The findings of this study may provide a theoretical basis for the environmentally sustainable and low-carbon management of greenhouse agriculture and its practical optimization.

## 2. Materials and Methods

### 2.1. Study Site

The experiment was conducted from March 2023 to December 2024 in an integrated water and fertilizer management demonstration greenhouse for protected vegetable production in Lvhua Village, Weiling Township, Qilihe District, Lanzhou, Gansu Province, China (36°2′23″ N, 103°42′39″ E). The experimental greenhouse was a typical east–west-oriented solar greenhouse constructed with brick–concrete walls and covered with polyvinyl chloride (PVC) film. An electric film-rolling system was installed to facilitate both roof and side ventilation. Temperature and humidity inside the greenhouse were regulated by adjusting the thermal insulation blanket and ventilation openings. The preceding crop was cucumber, and a one-month fallow period was maintained between cucumber cultivation and the establishment of the present experiment. The experimental site is located in a mid-temperate semi-arid climate zone at an elevation of 1871 m, with a mean annual temperature of 9.6 °C, a mean annual frost-free period of 190 d, and a mean annual precipitation of 344 mm, most of which occurs from July to September.

According to the international soil texture classification standard, the tested soil was classified as silt loam, consisting of 24.30% sand, 55.77% silt, and 19.93% clay. Before the initiation of the experiment, the physicochemical properties of the 0–20 cm soil layer were as follows: pH 7.61, electrical conductivity (EC) 302 μS cm^−1^, soil organic matter 16.91 g kg^−1^, alkali-hydrolyzable nitrogen 58.74 mg kg^−1^, available phosphorus 37.17 mg kg^−1^, available potassium 130.36 mg kg^−1^, and bulk density 1.28 g cm^−3^. Additional physicochemical properties of the experimental soil are presented in Table S1.

### 2.2. Experimental Materials

The tested crop was the tomato cultivar ‘Fenyan 734’ (produced by Shandong Chenghao Agricultural Technology Co., Ltd., Shouguang, China), which is suitable for autumn-extension cultivation in plastic tunnels and greenhouses. It is a premium large-fruited cultivar characterized by high yield and superior market quality. All tomato seedlings were transplanted at the three-true-leaf stage. The biogas slurry used in this study was obtained from Lanzhou Xinsu Ecological Energy Co., Ltd., Lanzhou, China. The company’s biogas project uses vegetables as the main fermentation feedstock. Prior to the experiment, the biogas slurry was allowed to stand for approximately 2 months, followed by solid–liquid separation. After its physicochemical properties had stabilized, it was filtered through four layers of 32-mesh gauze to remove larger suspended particles before use. The tested biochar was supplied by Liaoning Jinhefu Agricultural Development Co., Ltd., Anshan, China, and was produced from corn straw, corncobs, and peanut shells. The biochar was prepared under a pyrolysis temperature of 400–600 °C with a carbonization time of 4–6 h and was applied in powdered form. Before tomato planting, it was evenly applied to the soil surface and incorporated into the 0–30 cm tillage layer using a rotary tiller. The mineral fertilizers used in the experiment included urea (46% N; produced by Inner Mongolia Ordos United Chemical Co., Ltd., Ordos, China), calcium superphosphate (12% P_2_O_5_; produced by Hubei Fengle Ecological Fertilizer Co., Ltd., Zhongxiang, China), and potassium sulfate (52% K_2_O; produced by Yara Biotechnology Co., Ltd., Shouguang, China). The basic physicochemical properties of the biogas slurry and biochar are presented in Table S2.

### 2.3. Experimental Design

**Figure 1:**
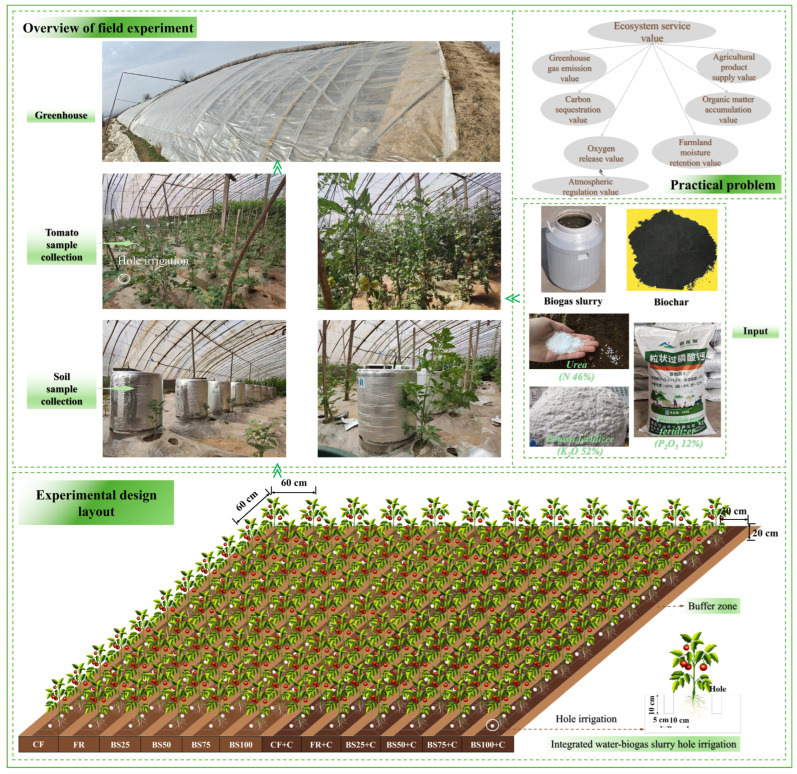
Schematic diagram of greenhouse experimental layout. For clarity, only one representative block of the randomized complete block design is shown.

According to the Chinese agricultural industry standard, technical code of practice for soil testing and formulated fertilization (NY/T 2911-2025), the target yield was set at 135,000 kg ha^−1^ based on the average tomato yield of the experimental greenhouse over the previous three years, representing a locally high yield level. According to the nutrient balance method of soil testing and formula fertilization (i.e., precision fertilization), the equivalent application amounts of N-P_2_O_5_-K_2_O for greenhouse tomato over the whole growing season were 380-180-500 kg ha^−1^ [20]. In the treatments with biogas slurry returning to the field, insufficient P_2_O_5_ and K_2_O were supplemented with mineral fertilizers (Table 1). Each experimental plot covered 126 m^2^ (7.0 m × 18.0 m), and a locally typical mulched planting pattern with alternating furrows and ridges was adopted (Figure 1). Single arch-shaped ridges were raised, with a ridge width of 0.3 m, a ridge height of 0.2 m, and both row and plant spacing of 0.6 m. Plastic film was placed between adjacent ridges to prevent lateral water seepage.

A randomized complete block design was adopted, with 12 treatment groups and three replicates for each treatment. The experimental results were expressed as the mean values of the three replicates. Under equivalent nutrient inputs of N, P_2_O_5_, and K_2_O among treatments (without considering differences in carbon inputs), the proportions of total applied N supplied by mineral fertilizers in each treatment were as follows: CF (conventional fertilization control, based on local farmer practice); FR (mineral fertilizer alone: soil testing-based formula fertilization, with mineral fertilizer N accounting for 100% of total applied N); BS25 (low-rate biogas slurry, with mineral fertilizer N accounting for 75% of total applied N); BS50 (medium-rate biogas slurry, with mineral fertilizer N accounting for 50% of total applied N); BS75 (high-rate biogas slurry, with mineral fertilizer N accounting for 25% of total applied N); BS100 (biogas slurry alone, with mineral fertilizer N accounting for 0% of total applied N); CF + C (conventional fertilization combined with biochar); FR + C (mineral fertilizer alone combined with biochar, with the total N from mineral fertilizers and biochar accounting for 100% of total applied N); BS25 + C (low-rate biogas slurry combined with biochar, with mineral fertilizer N accounting for 75% of total applied N); BS50 + C (medium-rate biogas slurry combined with biochar, with mineral fertilizer N accounting for 50% of total applied N); BS75 + C (high-rate biogas slurry combined with biochar, with mineral fertilizer N accounting for 25% of total applied N); and BS100 + C (biogas slurry alone combined with biochar, with mineral fertilizer N accounting for 0% of total applied N). Biochar was applied as a basal fertilizer at a rate of 45 t ha^−1^. This application rate was determined based on the optimal range for tomato yield improvement identified in a previous meta-analysis conducted by the research group and was considered suitable for greenhouse tomato production [21]. Referring to the growth characteristics and environmental requirements of tomato, and in combination with the long-term greenhouse tomato cultivation experience of the research group, the total fertilizer amount was allocated to basal fertilization, the seedling stage, the flowering stage, and the fruiting stage, accounting for 25%, 15%, 25%, and 35% of the total fertilizer amount, respectively. All other cultivation management practices were consistent with the local conventional planting pattern.

The tested biogas slurry was applied using the hole irrigation method [20]. Irrigation holes were arranged on the ridge top surface at positions 10 cm away from both sides of the crop, with a hole diameter of 5 cm and a depth of 10 cm. The irrigation frequency was set at once every 2 d. The irrigation amount was determined according to the cumulative evaporation measured by a φ20 cm evaporation pan placed at the same height as the crop canopy, and the evaporation measured at 8:00 a.m. over the interval between two irrigation events was used as the irrigation criterion to ensure identical water inputs among all treatments. The irrigation amount was calculated using Equation (1) [22]. To ensure identical total water inputs among all treatments, the net irrigation water input was determined by subtracting the volume of biogas slurry applied per irrigation event from the calculated irrigation amount.

(1) $$W=K_{p}SE_{p}$$

where *W* is the irrigation volume (mL), S is the wetted area (1800 cm^2^), *E_p_* is the cumulative evaporation recorded by a φ20 cm pan between two successive irrigation events (mm), and K_p_ is the crop-pan coefficient. To achieve water-saving control, a value of 0.8 was adopted in this study.

### 2.4. Sample Collection and Measurements

#### 2.4.1. Collection and Measurement of Tomato Samples

During the tomato seedling stage (spring–summer season: March–April; autumn–winter season: August–September), flowering stage (spring–summer season: April–May; autumn–winter season: September–October), and fruiting stage (spring–summer season: May–July; autumn–winter season: October–December), three plants were randomly selected from each replicate plot (i.e., nine plants per treatment), and the fresh weights of roots, stems, leaves, and fruits were measured separately. Subsequently, samples of each organ were placed into bags and transferred to an oven, where they were first heated at 105 °C for 60 min and then dried at 75 °C to constant weight. The dry matter weight was then measured and recorded.

At the tomato fruiting stage, the dried samples from each plant organ were ground into powder using an ultrafine grinder (FS-30C, Shandong Kaihui Machinery Equipment Co., Ltd., Jinan, China). A subsample of 50.0 mg was accurately weighed, wrapped in tin foil, and placed in the automatic sample tray of an elemental analyzer (Vario Macro CN, Elementar, Langenselbold, Germany) to determine plant carbon content. For yield determination, three tomato plants were randomly selected from each replicate plot (i.e., nine plants per treatment). The individual fruit weight was measured, and total fruit yield was recorded.

#### 2.4.2. Collection and Measurement of Soil Samples

Throughout the whole growing period of greenhouse tomato, five sampling points were randomly arranged in an “S”-shaped pattern in each treatment, and soil samples were collected by layer from depths of 0–10, 10–20, 20–30, 30–40, 40–50, and 50–60 cm. The five samples collected from the same soil layer were mixed, and soil moisture content was determined by the oven-drying method (105 °C, 12 h). In addition, at the tomato fruiting stage, soil samples from the 0–20 cm tillage layer were collected, and soil organic matter was determined using the potassium dichromate volumetric method with external heating (ferrous sulfate titration) [20].

#### 2.4.3. Collection and Measurement of Soil Greenhouse Gases

Soil gases were collected in situ using a static dark chamber system consisting of a chamber and a base (manufactured by Suzhou Scientific Research Partner Co., Ltd., Suzhou, China). The chamber was made of 5 mm thick polyvinyl chloride (PVC) and measured 31.5 cm in diameter and 40 cm in height. Its outer surface was wrapped with foam and reflective thermal insulation film to reduce temperature fluctuations inside the chamber. A small fan was installed at the top of the chamber to ensure uniform gas mixing. The circular base was made of stainless steel and was installed on the day of tomato transplanting between two seedlings in the center of the ridge, where it remained until harvest. The base covered half of the root zone of the two plants and was used to determine greenhouse gas fluxes from the “roots + soil” system. The base was inserted 8–10 cm into the soil, and a 3 cm deep groove was formed at its upper edge for chamber placement. During sampling, water was added to the groove to create a seal and isolate the chamber from the external environment. Gas sampling began 10 d after transplanting. Based on previous research experience, each sampling event was conducted between 09:30 and 10:30. Gas samples were collected on days 1, 3, and 7 after the tomato seedling recovery stage, and thereafter once every 14 d throughout the remaining growth period. Gas samples were collected four times using a syringe fitted with a three-way stopcock. At 0, 10, 20, and 30 min after chamber closure, 100 mL of gas was collected into aluminum foil sampling bags (E-Switch, Shanghai Shenyuan Scientific Instrument Co., Ltd., Shanghai, China), which were then sealed and stored at room temperature until analysis.

The concentrations of CO_2_, N_2_O, and CH_4_ were determined using a gas chromatograph (Agilent Technologies 7890A GC System, Santa Clara, CA, USA). CO_2_ and CH_4_ concentrations were determined using a flame ionization detector (FID), with a column temperature of 80 °C, a detector temperature of 200 °C, N_2_ as the carrier gas at a flow rate of 40 mL min^−1^, H_2_ as the fuel gas at a flow rate of 35 mL min^−1^, and air as the oxidant gas at a flow rate of 350 mL min^−1^. N_2_O concentration was determined using an electron capture detector (ECD), with a column temperature of 80 °C, a detector temperature of 320 °C, and argon–methane as the carrier gas at a flow rate of 30 mL min^−1^. Greenhouse gas emission fluxes were calculated using the following equation [23]:

(2) $$F=\rho \times h\times \frac{dc}{dt}\times \frac{273}{\left(273+T\right)}\times 60$$

where *F* represents the greenhouse gas flux (mg m^−2^ h^−1^), *ρ* is the gas density under standard conditions, with values of 1.964, 0.714, and 1.977 g L^−1^ for N_2_O, CH_4_, and CO_2_, respectively, *h* is the effective height of the chamber during sampling (m), *dc/dt* is the rate of change in gas concentration inside the chamber (ppm min^−1^), *T* is the average air temperature inside the chamber during sampling (°C), and 60 is the conversion factor from minutes to hours.

(3) $$C=\Sigma \left(\frac{F_{i}+F_{i+1}}{2}\times d\times 24\right)/100$$

where *C* represents the cumulative greenhouse gas emissions (kg ha^−1^), *F_i_* and *F_i+_*_1_ denote the gas fluxes at two consecutive sampling times (mg m^−2^ h^−1^), *d* is the interval in days between the two sampling dates, and 24 is the conversion factor from hours to days. During each gas sampling event, the temperature inside the chamber was measured using a red alcohol thermometer inserted at the top of the chamber.

### 2.5. Net Ecosystem Carbon Balance of the Farmland Ecosystem

Based on the method reported in the literature [24], the net ecosystem carbon balance (*N*_ECB_) of the farmland ecosystem was estimated according to the principle of balancing carbon inputs and outputs, and the equations were as follows:

(4) $$N_{\mathrm{ECB}}=Z_{\mathrm{NPP}}+C_{amendment}-C_{h}-C_{s}$$

(5) $$Z_{\mathrm{NPP}}=F_{\mathrm{NPP}}+L_{\mathrm{NPP}}+S_{\mathrm{NPP}}+R_{\mathrm{NPP}}$$

where *N*_ECB_ is the net ecosystem carbon balance of the farmland ecosystem (t ha^−1^); *Z*_NPP_ is the net primary productivity-fixed carbon (NPP-fixed carbon, t ha^−1^); *F*_NPP_, *L*_NPP_, *S*_NPP_, and *R*_NPP_ represent the carbon contents in tomato fruits, leaves, stems, and roots, respectively (root carbon refers only to carbon stored in living root biomass and excludes root exudates and dead root residues, t ha^−1^); *C*_amendment_ is fertilizer carbon input (including the accounted carbon inputs derived from mineral fertilizers, biogas slurry, and biochar, t ha^−1^); *C*_h_ is fruit carbon export, i.e., yield carbon (t ha^−1^); and *C*_s_ is soil carbon output, including CO_2_ and CH_4_ but excluding dissolved organic carbon losses (t ha^−1^). The accounted carbon input from biochar was estimated according to the proportion of nitrogen supplied by biochar relative to the total applied nitrogen in each treatment. The corresponding biochar application rate was then calculated and multiplied by the biochar carbon concentration (48.19%) to determine the biochar-derived carbon input. This carbon input was fully incorporated into the carbon balance of the current growing season.

### 2.6. Ecosystem Service Value

The market value method was used to estimate the agricultural product supply value of the farmland ecosystem [19,25], and the equation was as follows:

(6) $$V_{1}=Y\times P-I_{C}$$

where *V*_1_ is the agricultural product supply value (CNY ha^−1^); *Y* is the tomato yield (t ha^−1^); *P* is the market price of tomato fruits (CNY kg^−1^); and *I_C_* is the cost input for tomato production (CNY ha^−1^), which mainly consisted of production material inputs, while machinery inputs and labor costs were not included.

In this study, machinery inputs and labor costs were not included in the calculation of agricultural product supply value for the following four reasons. First, the greenhouse experimental plots were limited in size, and the degree of mechanized operation was low; therefore, the associated costs were relatively low and difficult to allocate accurately. Second, the core purpose of the experiment was to control variables so as to focus on evaluating the production performance of specific agricultural inputs or agronomic practices. Treating machinery and labor inputs as fixed background conditions was conducive to clearly revealing differences in biological output among treatments. Third, labor in small-scale scientific research management was mainly undertaken by researchers or students, whose work characteristics differed substantially from those of market-based labor hours, resulting in ambiguous cost boundaries that were difficult to quantify. Fourth, under the condition that machinery and labor inputs were basically consistent among treatments, omission of these costs did not affect the relative comparison among treatments and also helped improve the generality and comparability of the research results.

The organic matter accumulation value of the farmland ecosystem was evaluated using the soil organic matter retention method and the opportunity cost method, and the equation was as follows:

(7) $$V_{2}=S_{h}\times O_{M}\times r\times P_{O}$$

where *V*_2_ is the organic matter accumulation value of farmland (CNY ha^−1^); *S_h_* is the soil layer thickness, taken as 20 cm; *O_M_* is the soil organic matter content (g kg^−1^); *r* is the field soil bulk density (g cm^−3^); and *P_O_* is the price of organic matter (CNY t^−1^), which was converted from the opportunity cost price of fuelwood (51.3 CNY t^−1^) based on a fuelwood conversion ratio of 2:1.

The atmospheric regulation function of farmland ecosystems mainly involves the positive effects of carbon sequestration and oxygen release, as well as the negative effects of greenhouse gas emissions. Based on the principle of photosynthesis, namely that for every 1 kg of dry matter produced by plants, 1.63 kg of CO_2_ can be fixed and 1.19 kg of O_2_ can be released, the carbon sequestration value and oxygen release value of the farmland ecosystem were further calculated using the afforestation cost method [19].

The carbon sequestration value and oxygen release value of crops were calculated using Equations (8) and (9), respectively:

(8) $$V_{3}=Z_{\mathrm{NPP}}\times 1.63\times P_{C}\times \frac{3}{11}$$

(9) $$V_{4}=Z_{\mathrm{NPP}}\times 1.19\times P_{1}$$

where *V*_3_ is the carbon sequestration value (CNY ha^−1^); *P_C_* is the value of fixed CO_2_, taken as 0.63 CNY kg^−1^; 3/11 is the mass fraction of C in CO_2_; *V*_4_ is the oxygen release value (CNY ha^−1^); and *P*_1_ is the value of oxygen production, taken as 0.38 CNY kg^−1^, which was the mean of the values calculated by the industrial oxygen production method and the afforestation cost method.

The greenhouse gas emission value was calculated by first converting N_2_O and CH_4_ emissions into CO_2_ equivalents based on global warming potential (GWP), followed by conversion into carbon equivalents (kg C ha^−1^). In addition, CO_2_ emissions associated with the production and transportation of biochar applied in each treatment were estimated using the corresponding emission factors, converted into carbon equivalents, and incorporated into the total greenhouse gas emission accounting [26]. The greenhouse gas emission value was then quantified using the carbon sequestration afforestation cost method. GWP was assessed over a 100-year time horizon. On a unit mass basis, the GWP values of CH_4_ and N_2_O were 27.9 and 273.0 times that of CO_2_, respectively [23]. The equations were as follows:

(10) $$V_{5}=GWP\times P_{C}\times \frac{3}{11}$$

(11) $$GWP=f_{CO_{2}}+273.0f_{N_{2}O}+27.9f_{CH_{4}}+f_{biochar}$$

where *V*_5_ is the greenhouse gas emission value (CNY ha^−1^); GWP is the global warming potential (kg ha^−1^); and *f*_CO2_, *f*_N2O_, *f*_CH4_ and *f*_biochar_ represent the total cumulative emissions of CO_2_, N_2_O, CH_4_, and biochar respectively (kg ha^−1^).

For the farmland moisture retention value, no monetary accounting was conducted in this study; instead, only changes in soil moisture content during different growth stages were recorded, mainly for the following two reasons. One reason is that, at the plot experimental scale, the valuation of hydrological services depends heavily on assumptions related to boundary delineation, parameter extrapolation, and value equivalence, which would introduce substantial uncertainty and subjectivity, thereby reducing the objectivity and comparability of the research conclusions. Another reason is that, as a directly observable core physical parameter, soil moisture content can directly support the primary analysis of agricultural product supply value in this study by reflecting the actual effect of water on crop growth, while also providing reliable baseline data for broader subsequent studies on ecological value. This approach was adopted to avoid forced monetization in a methodological area that remains insufficiently mature, thereby ensuring the robustness and scientific validity of the study conclusions.

### 2.7. VIKOR Evaluation Model Based on AHP-CRITIC Combination Weighting

To address the limitations of a single weighting method in multi-criteria decision-making, a VIKOR evaluation model based on AHP-CRITIC combination weighting was constructed in this study [27]. The analytic hierarchy process (AHP) was used to reflect decision-makers’ subjective judgments regarding the relative importance of each evaluation indicator, where subjective weights refer to weights determined based on expert knowledge and decision preferences. However, it may be influenced by differences in expert experience and preferences, resulting in a certain degree of subjectivity. The CRITIC method was used to assign objective weights according to the degree of variation in indicator data and the conflict among indicators, thereby effectively extracting information from the data itself. Objective weights refer to weights derived from the statistical characteristics of the observed data rather than expert judgment. However, it relies heavily on sample data and is less capable of reflecting decision-makers’ actual preferences. To integrate subjective judgments and objective data characteristics, AHP and CRITIC were combined in this study to determine indicator weights, thereby improving the scientific rigor, rationality, and robustness of the weighting results.

Specifically, the AHP method employed the Saaty 1–9 scale to construct the judgment matrix and calculate subjective weights. Consistency testing was subsequently performed, and the consistency ratio (CR) was less than 0.10, indicating acceptable consistency. The consistency test and the combination of AHP and CRITIC weighting methods were used to improve the reliability of the evaluation results. Prior to CRITIC weighting, the original data were normalized using the min–max normalization method. The standard deviation of each indicator (representing contrast intensity) and the Pearson correlation coefficient between indicators (representing conflict) were then calculated. A stronger positive correlation between indicators indicated greater information redundancy and consequently a lower indicator weight. Objective weights were subsequently obtained based on these measures. The combined weights were calculated using the multiplicative normalization formula (Equation (12)):

(12) $$W_{j}=\frac{AHP_{j}\times CRITIC_{j}}{\sum_{j=1}^{n}\left(AHP_{j}\times CRITIC_{j}\right)}$$

where *W_j_* is the combined weight, *AHP_j_* is the subjective weight, *CRITIC_j_* is the objective weight, and *n* is the number of evaluation indicators.

After the combination weights had been obtained, the VIKOR method was further used to comprehensively evaluate and rank the alternative schemes. Using the ideal solution and the negative ideal solution as references, this method measures the proximity of each alternative to the ideal scheme by calculating the group utility value (*S_i_*), the individual regret value (*R_i_*), and the comprehensive compromise indicator (*Q_i_*), and then determines the ranking order of the alternatives accordingly. The VIKOR method can seek a balance between maximizing overall group utility and minimizing individual regret, thereby obtaining the best compromise solution that accounts for both overall benefit and local satisfaction. Therefore, the evaluation framework integrating AHP-CRITIC combination weighting with the VIKOR method can provide a more scientific and reliable basis for decision-making in the selection of optimal schemes under multi-criteria conditions.

### 2.8. Data Processing

Microsoft Excel 2016 was used for data processing. Origin 2021 was used for figure preparation and correlation analysis (*p* < 0.05). IBM SPSS Statistics 25 was used for one-way analysis of variance, and the Waller-Duncan test was used for post hoc multiple comparisons, with the significance level set at 0.05. The hierarchical model diagram for the comprehensive evaluation of ecosystem service value in the greenhouse farmland ecosystem was produced using Visio Plan 2.

## 3. Results

### 3.1. Effects of Biogas Slurry–Biochar–Mineral Fertilizer Co-Application on Soil Moisture Content and Organic Matter in Greenhouse Tomato Soil

**Figure 2:**
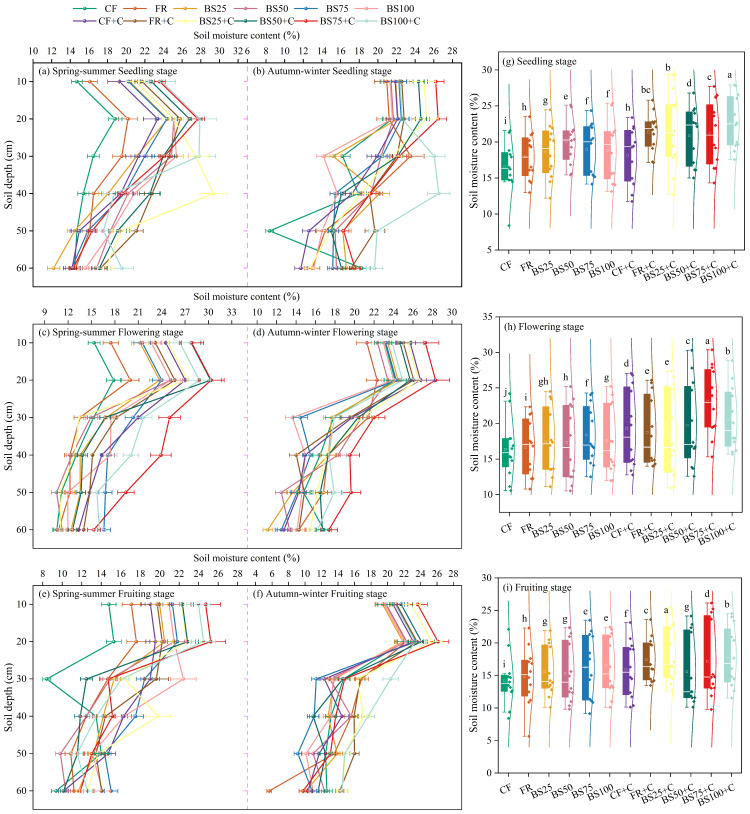
Effects of different biogas slurry–biochar–mineral fertilizer co-application treatments on soil moisture content. Panels (**a**–**f**) present soil moisture content at various soil depths across different growth stages during the spring–summer and autumn–winter seasons. Error bars represent standard deviations (SD). Panels (**g**,**h**,**i**) show soil moisture content at different growth stages. Different lowercase letters within the same growth stage indicate significant differences among treatments (*p* < 0.05).

As shown in Figure 2a–f, soil moisture content in all treatments generally exhibited a trend of first increasing and then decreasing along the vertical profile at different growth stages, with the most pronounced increase occurring in the 10–20 cm soil layer. Significant differences in soil moisture content were observed among treatments (*p* < 0.05; Figure 2g–i). Under equal inputs of N, P_2_O_5_, and K_2_O, soil moisture content across all treatments showed a pattern of “increasing first and then decreasing” over the whole growing period, reaching a peak at the flowering stage. Specifically, compared with the treatments without biochar application (CF-BS100), biochar co-application (CF + C-BS100 + C) significantly increased the mean soil moisture content at both the seedling stage and the flowering stage (seedling stage: increases of 4.69–29.34% in the spring–summer season and 1.79–37.34% in the autumn–winter season; flowering stage: increases of 1.98–40.66% in the spring–summer season and 0.89–28.95% in the autumn–winter season), whereas the increasing trend weakened by the fruiting stage. Negative growth was even observed in BS75 + C and BS100 + C in the spring–summer season and in CF + C in the autumn–winter season. The extreme values of soil moisture content at different growth stages were as follows: at the seedling stage in the spring–summer season, BS25 + C showed the highest value (23.36%), whereas CF showed the lowest value (15.76%); at the seedling stage in the autumn–winter season, BS50 + C showed the highest value (22.87%), whereas BS50 showed the lowest value (16.65%); at the flowering stage in the spring–summer season, BS75 + C showed the highest value (23.65%), whereas CF showed the lowest value (13.87%); at the flowering stage in the autumn–winter season, BS75 + C showed the highest value (22.36%), whereas BS75 showed the lowest value (17.34%); at the fruiting stage in the spring–summer season, BS75 showed the highest value (18.08%), whereas CF showed the lowest value (12.56%); and at the fruiting stage in the autumn–winter season, BS100 + C showed the highest value (18.60%), whereas BS75 showed the lowest value (14.49%).

As shown in Table 2, compared with the CF-BS100 treatments, the CF + C-BS100 + C treatments significantly increased soil organic matter content in the 0–20 cm soil layer. On a concentration basis (g kg^−1^), the increases ranged from 13.55% to 45.07% in the spring–summer season and from 20.10% to 81.15% in the autumn–winter season. On a stock basis (t ha^−1^), compared with the control, biochar-amended treatments showed increases ranging from 8.15% to 40.16% in the spring–summer season and from 19.94% to 79.48% in the autumn–winter season, further confirming that biochar application had a stable and significant positive effect on soil organic matter storage. Regarding extreme values, the highest values in both growing seasons were observed in the BS75 + C treatment (72.26 t ha^−1^ in spring–summer and 71.93 t ha^−1^ in autumn–winter), whereas the lowest values occurred in different treatments across seasons, with FR in the spring–summer season (47.31 t ha^−1^) and BS100 in the autumn–winter season (39.36 t ha^−1^). Overall, soil organic matter stock under different treatments showed a similar pattern across the two growing seasons, and biochar combined with mineral fertilizer treatments exhibited higher soil organic matter accumulation capacity.

### 3.2. Effects of Biogas Slurry–Biochar–Mineral Fertilizer Co-Application on Tomato Yield and Biomass

As shown in Table 2, under the same inputs of N, P_2_O_5_, and K_2_O, BS75 + C produced the highest tomato yield, reaching 152.39 t ha^−1^ in the spring–summer season and 150.29 t ha^−1^ in the autumn–winter season, which were significantly higher than those of the other treatments (*p* < 0.05), with increases of 6.86–48.56% and 7.20–50.02%, respectively. BS25 ranked second in tomato yield, with values of 142.60 t ha^−1^ in the spring–summer season and 140.20 t ha^−1^ in the autumn–winter season. Overall, at the same substitution ratio, biochar co-application generally increased greenhouse tomato yield, with increases ranging from 1.85% to 35.39% in the spring–summer season and from 2.12% to 36.53% in the autumn–winter season.

Table 2 also shows that CF + C produced the highest biomass, reaching 36.19 t ha^−1^ in the spring–summer season and 35.57 t ha^−1^ in the autumn–winter season, and these values were significantly higher than those of the other treatments (*p* < 0.05), with increases of 0.21–76.35% and 0.21–78.75%, respectively. BS50 ranked second in biomass, with values of 36.11 t ha^−1^ in the spring–summer season and 35.49 t ha^−1^ in the autumn–winter season, whereas FR showed the lowest biomass, with values of 20.52 t ha^−1^ in the spring–summer season and 19.90 t ha^−1^ in the autumn–winter season. Overall, at the same substitution ratio, biochar co-application significantly increased biomass, with increases ranging from 3.14% to 28.24% in the spring–summer season and from 3.23% to 29.05% in the autumn–winter season. These results indicate that an appropriate substitution of mineral fertilizer with biogas slurry contributes to increased tomato yield and biomass, whereas the co-application of biogas slurry and biochar further enables a higher substitution ratio by enhancing nutrient and water retention in the root-zone soil.

### 3.3. Effects of Biogas Slurry–Biochar–Mineral Fertilizer Co-Application on Cumulative Soil Carbon Emissions

As shown in Table 2, compared with the autumn–winter season, cumulative soil CO_2_ emissions increased in all treatments during the spring–summer season, with increases ranging from 5.76% to 14.60%. BS100 + C showed the highest cumulative soil CO_2_ emissions, reaching 11.53 t ha^−1^ in the spring–summer season and 10.82 t ha^−1^ in the autumn–winter season, which were significantly higher than those of the other treatments (*p* < 0.05), with increases of 0.63–40.65% and 4.25–45.25%, respectively. BS50 + C ranked second in cumulative soil CO_2_ emissions, with values of 11.46 t ha^−1^ in the spring–summer season and 10.38 t ha^−1^ in the autumn–winter season, whereas CF showed the lowest cumulative soil CO_2_ emissions, with values of 8.20 t ha^−1^ in the spring–summer season and 7.45 t ha^−1^ in the autumn–winter season.

Differences in cumulative soil CH_4_ emissions were also observed among treatments, and overall, the soil acted as a CH_4_ sink. Negative values indicate net CH_4_ uptake by the soil, suggesting that CH_4_ oxidation by aerobic methanotrophs exceeded CH_4_ production by anaerobic methanogens, resulting in a negative cumulative flux. By the end of the growing period, cumulative soil CH_4_ emissions ranged from −2.68 to −0.18 kg ha^−1^ in the spring–summer season and from −2.75 to −0.21 kg ha^−1^ in the autumn–winter season, showing a clear uptake pattern. Specifically, BS100 showed the highest cumulative soil CH_4_ emissions, reaching −0.18 kg ha^−1^ in the spring–summer season and −0.21 kg ha^−1^ in the autumn–winter season, which were significantly higher than those of the other treatments (*p* < 0.05). FR ranked second in cumulative soil CH_4_ emissions, with values of −0.33 kg ha^−1^ in the spring–summer season and −0.35 kg ha^−1^ in the autumn–winter season, whereas CF + C showed the lowest cumulative soil CH_4_ emissions, with values of −2.68 kg ha^−1^ in the spring–summer season and −2.75 kg ha^−1^ in the autumn–winter season.

### 3.4. Effects of Biogas Slurry–Biochar–Mineral Fertilizer Co-Application on the Net Ecosystem Carbon Balance of the Greenhouse Farmland Ecosystem

The net ecosystem carbon balance (*N*_ECB_) of the greenhouse tomato farmland ecosystem and its component terms in the spring–summer and autumn–winter seasons are shown in Table 3. NPP-fixed carbon was the dominant carbon input component of the farmland ecosystem (Figure S1), ranging from 11.00 to 18.09 t ha^−1^ in the spring–summer season and from 9.71 to 17.08 t ha^−1^ in the autumn–winter season across treatments. Among them, BS75 + C showed the highest NPP-fixed carbon, reaching 18.09 t ha^−1^ in the spring–summer season and 17.08 t ha^−1^ in the autumn–winter season, which were significantly higher than those of the other treatments (*p* < 0.05), with increases of 2.88–64.47% and 0.91–75.93%, respectively. Fertilizer carbon input ranged from 0.1652 to 0.3382 t ha^−1^ across treatments. Among them, BS100 showed the highest fertilizer carbon input (0.3382 t ha^−1^), followed by BS100 + C (0.3353 t ha^−1^), whereas FR showed the lowest fertilizer carbon input (0.1652 t ha^−1^).

In terms of carbon output, soil carbon output (CO_2_ and CH_4_) was the dominant carbon source component of the system, followed by fruit carbon export. Specifically, BS100 + C showed the highest soil carbon output, reaching 11.50 t ha^−1^ in the spring–summer season and 10.79 t ha^−1^ in the autumn–winter season, which were significantly higher than those of the other treatments (*p* < 0.05), with increases of 0.93–40.97% and 4.62–45.64%, respectively. Compared with the autumn–winter season, soil carbon output increased in all treatments during the spring–summer season, with increases ranging from 5.78% to 14.66%. BS75 + C showed the highest fruit carbon export, reaching 2.26 t ha^−1^ in the spring–summer season and 2.22 t ha^−1^ in the autumn–winter season, which were significantly higher than those of the other treatments (*p* < 0.05), with increases of 13.38–203.41% and 13.61–206.60%, respectively. Compared with the autumn–winter season, fruit carbon export increased in all treatments during the spring–summer season, with increases ranging from 1.74% to 2.81%. Overall, although BS75 + C showed the highest fruit carbon export, it also had the highest NPP-fixed carbon and therefore exhibited the highest *N*_ECB_, reaching 6.30 t ha^−1^ in the spring–summer season and 6.34 t ha^−1^ in the autumn–winter season. These results indicate that the BS75 + C pattern can effectively enhance the carbon sink capacity of farmland while ensuring tomato yield, thereby providing a feasible pathway for the development of high-yield and low-carbon agriculture.

### 3.5. Effects of Biogas Slurry–Biochar–Mineral Fertilizer Co-Application on Agricultural Product Supply Value in Greenhouse Production

Within the accounting framework of this study, agricultural product supply value could be regarded, both numerically and conceptually, as an expression of the economic benefits of agricultural production (Table 4). The results showed that agricultural product supply value ranged from 402,813.91 to 634,041.87 CNY ha^−1^ in the spring–summer season and from 393,412.21 to 623,182.17 CNY ha^−1^ in the autumn–winter season across treatments. Among the components used to calculate agricultural product supply value, yield revenue accounted for the largest proportion, followed by the cost of biochar (Figure S2). BS25 exhibited the highest agricultural product supply value in both experimental seasons, reaching 634,041.87 CNY ha^−1^ and 623,182.17 CNY ha^−1^, respectively. Both values were significantly higher than those of the other treatments (*p* < 0.05), representing increases of 11.15% to 57.40% and 11.37% to 58.40%, respectively. By contrast, the agricultural product supply value of BS75 + C was at an intermediate level, with values of 488,710.49 CNY ha^−1^ in the spring–summer season and 479,308.79 CNY ha^−1^ in the autumn–winter season. Overall, the agricultural product supply value of the biochar-amended treatments was generally lower than that of the corresponding treatments without biochar, with decreases of 0.74% to 34.05% and 0.42% to 34.41%, respectively. This may be related to the relatively high input cost of biochar and the weakened yield-enhancing effect under low-temperature conditions. In addition, treatments with a higher substitution ratio of biogas slurry for mineral fertilizers, such as BS100, did not produce a higher agricultural product supply value, indicating that complete substitution of mineral fertilizer with biogas slurry was not the optimal application strategy.

Overall, under the conditions of this experiment, two fertilization strategies with practical value could be adopted to achieve different production objectives. If maximizing economic benefits, that is, agricultural product supply value, is the primary objective, 25% biogas slurry substitution for mineral fertilizer is preferred. If maximizing fruit yield is the primary objective, a strategy of 75% biogas slurry combined with biochar to substitute for mineral fertilizer is more appropriate.

### 3.6. Effects of Biogas Slurry–Biochar–Mineral Fertilizer Co-Application on Ecosystem Service Value in the Greenhouse Farmland Ecosystem

Ecosystem service value refers to an economic valuation framework used to quantify the various benefits provided by ecosystems that support human survival. In this study, the evaluated ecosystem service value included agricultural product supply value, organic matter accumulation value, and atmospheric regulation value, the latter comprising carbon sequestration value, oxygen release value, and greenhouse gas emission value. Among these components, only greenhouse gas emissions showed a negative value, and the changes in emissions over the entire growth period in different experimental seasons are shown in Figure S3. The results showed that ecosystem service value ranged from 411,604.51 to 641,606.83 CNY ha^−1^ in the spring–summer season and from 401,746.49 to 629,987.37 CNY ha^−1^ in the autumn–winter season across treatments (Table 5). Among the treatments, BS25 showed the highest ecosystem service value, reaching 641,606.83 CNY ha^−1^ in the spring–summer season and 629,987.37 CNY ha^−1^ in the autumn–winter season. Both values were significantly higher than those of the other treatments (*p* < 0.05), representing increases of 10.12% to 55.88% and 10.34% to 56.81%, respectively. BS50 ranked second, with ecosystem service values of 582,641.40 CNY ha^−1^ in the spring–summer season and 570,930.72 CNY ha^−1^ in the autumn–winter season. By contrast, the ecosystem service value of BS75 + C was at an intermediate level, with values of 501,551.08 CNY ha^−1^ and 491,577.09 CNY ha^−1^ in the spring–summer and autumn–winter seasons, respectively.

For organic matter accumulation value, the values ranged from 1213.44 to 1853.57 CNY ha^−1^ in the spring–summer season and from 1009.57 to 1844.88 CNY ha^−1^ in the autumn–winter season across treatments. Among the treatments, BS75 + C showed the highest organic matter accumulation value, followed by BS50 + C. By contrast, BS25 showed a relatively moderate level of organic matter accumulation value, with values of 1286.59 CNY ha^−1^ in the spring–summer season and 1288.91 CNY ha^−1^ in the autumn–winter season. For atmospheric regulation value, the values ranged from 5769.36 to 10,987.03 CNY ha^−1^ in the spring–summer season and from 5345.10 to 10,423.42 CNY ha^−1^ in the autumn–winter season across treatments. Among the treatments, BS75 + C showed the highest atmospheric regulation value, whereas BS25 showed the lowest.

Overall, under different fertilization strategies, ecosystem service value and individual service values showed differentiated patterns. BS25, representing 25% substitution of mineral fertilizer with biogas slurry, achieved the highest ecosystem service value because of its highest agricultural product supply value. By contrast, BS75 + C, representing 75% substitution of mineral fertilizer with biogas slurry combined with biochar, performed best in ecological regulation services because of its superior soil carbon sequestration and atmospheric regulation functions. These results indicate that maximizing economic benefits and maximizing ecological functions correspond to different optimization pathways. Therefore, fertilization strategies should be selected according to specific practical objectives, such as short-term income enhancement or long-term carbon sequestration.

### 3.7. Appropriate Co-Application Pattern of Biogas Slurry, Biochar, and Mineral Fertilizer Based on the VIKOR Evaluation Model

**Figure 3:**
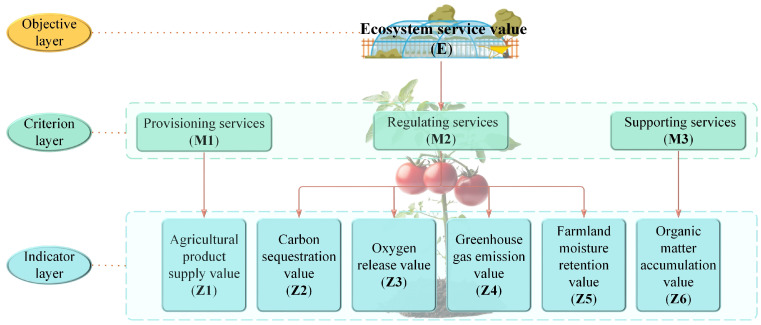
Hierarchical model for the comprehensive evaluation of ecosystem service value in the greenhouse farmland ecosystem.

According to the service types covered by ecosystem service value in this study, namely provisioning services, regulating services, and supporting services, an evaluation index system composed of individual ecosystem service values was constructed, including agricultural product supply value, atmospheric regulation value, farmland moisture retention value, and organic matter accumulation value. The combination-weighting VIKOR method was then used for multi-objective comprehensive evaluation. By constructing a hierarchical model for the comprehensive evaluation of ecosystem service value in the greenhouse farmland ecosystem (Figure 3), the target layer (E) was divided into three criterion layers, namely provisioning services (M1), regulating services (M2), and supporting services (M3). A total of six individual indicators under these criterion layers constituted the indicator layer. The analytic hierarchy process and the entropy weight method were used to calculate the subjective and objective weights, respectively, and the combination weights of the individual indicators were determined using a game theory-based combination-weighting method. The results are presented in Table 6.

The group utility value (*S_i_*), individual regret value (*R_i_*), and comprehensive compromise indicator (*Q_i_*) for different substitution ratios of biogas slurry combined with biochar for mineral fertilizer are shown in Table 6, where a smaller *Q_i_* value indicates a better scheme. Among all treatments, BS75 + C had the smallest *Q_i_* value, at 0.0444, indicating the best overall evaluation. BS50 ranked second, with a *Q_i_* value of 0.0744. FR had the largest *Q_i_* value, at 0.9728, indicating the poorest comprehensive performance. These results indicate that the combined application of a high proportion of biogas slurry and biochar as a partial substitute for mineral fertilizer can better coordinate provisioning, regulating, and supporting services in greenhouse farmland, thereby improving overall ecosystem service performance. The medium-rate biogas slurry substitution treatment also showed relatively good overall performance, whereas the mineral fertilizer-only treatment showed the poorest overall performance. Therefore, under the conditions of this experiment, the treatment combining a high proportion of biogas slurry with biochar, in which mineral fertilizer N accounted for 25% of the total N application, may be considered a suitable strategy for green and low-carbon management in greenhouse agriculture.

### 3.8. Relationship Between Ecosystem Service Value and Comprehensive Compromise Indicator Under Different Seasonal Conditions

**Figure 4:**
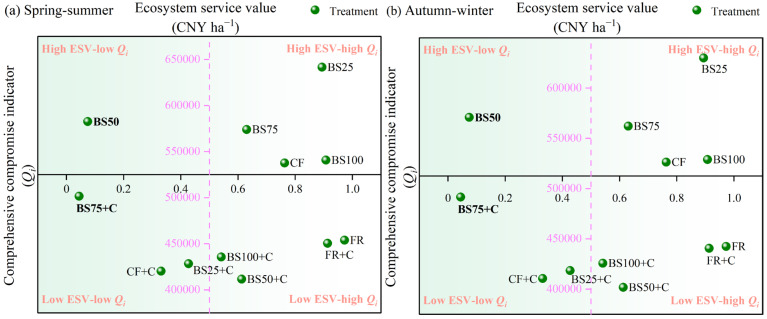
Relationship between ecosystem service value (ESV) and comprehensive compromise indicator (*Q_i_*) under different treatments. The treatment combinations are divided into four categories based on the four quadrants, categorizing them into high ESV/low ESV and high *Q_i_*/low *Q_i_*.

Under different seasonal conditions (Figure 4), BS50 (medium-rate biogas slurry: mineral fertilizer N accounts for 50% of total N applied) exhibited lower comprehensive compromise indicator (*Q_i_*) values and higher ecosystem service values (ESVs) in both spring–summer and autumn–winter seasons, showing a favorable overall evaluation. BS75 + C (high-rate biogas slurry combined with biochar: mineral fertilizer N accounting for 25% of total applied N) demonstrated the smallest *Q_i_* value and moderate ESV, indicating that the incorporation of biochar helps optimize fertilization performance. BS25 (low-rate biogas slurry: mineral fertilizer N accounting for 75% of total applied N) exhibited higher ESV and *Q_i_* values in both spring–summer and autumn–winter seasons, suggesting a poor overall evaluation effect in different seasons. Although ESV was high, the similarly high *Q_i_* value indicated that BS25 is unsuitable as a long-term fertilization strategy. In contrast, CF (conventional fertilization control) and FR (mineral fertilizer-only treatment) showed higher *Q_i_* values and lower ESV, reflecting poorer overall performance. Although BS100 (biogas slurry-only treatment) showed moderate ESV, its comprehensive evaluation effect was relatively poor. Overall, BS50 and BS75 + C are effective in enhancing ecological benefits and optimizing fertilization strategies, making them suitable for long-term agricultural sustainability and environmental protection goals. BS25 showed poor comprehensive evaluation effects and is thus unsuitable for long-term fertilization schemes. CF and FR should be avoided for long-term use to prevent negative impacts on soil quality.

## 4. Discussion

### 4.1. Effects of Biogas Slurry–Biochar–Mineral Fertilizer Co-Application on Soil Organic Matter and Production of Greenhouse Tomato

As two important organic materials, biogas slurry and biochar can effectively achieve organic substitution in practical application, thereby reducing mineral fertilizer use while ensuring crop growth, increasing yield, and promoting the green and ecological development of agriculture [28]. The results of the present study showed that, under equal nutrient input conditions, the treatment with the highest biomass (CF + C) did not achieve the highest yield. Instead, the highest yield was obtained under the treatment in which biogas slurry combined with biochar substituted for 75% mineral fertilizer N (BS75 + C), and BS25 + C showed the second-highest yield. At the same biogas slurry substitution ratio, biochar application consistently increased greenhouse tomato yield (Table 2). Comprehensive analysis suggested that the differences in tomato production mainly arose from the following three aspects.

One possible explanation is that different fertilization patterns exerted different regulatory effects on crop source–sink allocation. Crop economic yield depends not only on the total amount of photosynthates produced, namely source strength, but also on the allocation efficiency of assimilates to harvest organs, namely sink strength and allocation coordination. Under conventional fertilization combined with biochar, the continuous and excessive supply of readily available N may have excessively stimulated vegetative growth, which could have led to deterioration of canopy structure and restricted assimilate allocation to fruits, thereby reducing the harvest index [29,30]. Another important factor is that the substitution ratio of biogas slurry needs to be synergistically optimized with biochar application to achieve the best effect on tomato growth. Previous studies have shown that, in the absence of biochar application, an appropriate substitution of biogas slurry for mineral fertilizer can promote the growth of greenhouse tomatoes and improve yield and quality. However, excessive substitution may inhibit root development because of salt accumulation and reduced soil aeration, thereby causing leaf chlorosis and wilting and ultimately reducing yield [31]. The results of the present study showed that the BS75 + C treatment maintained the highest soil moisture content during the flowering stage (23.65% in the spring–summer season and 22.36% in the autumn–winter season), which was conducive to nutrient uptake and yield formation. Consequently, tomato yield was highest under the condition of high-rate biogas slurry combined with biochar, indicating that biochar may broaden the suitable substitution range of biogas slurry by improving soil structure and alleviating the potential negative effects of biogas slurry. A further explanation is that the yield-enhancing effect of biochar depends on the nutrient management system. When biochar was co-applied in all treatments, its enhancement mechanism depended on fertilizer type. Under conventional fertilization, biochar mainly exerted physical functions related to nutrient and water retention [32]. By contrast, under high-rate biogas slurry combined with biochar, biochar showed a pronounced combined enhancement effect on the accumulation of soil organic matter supplied by biogas slurry. Compared with the initial soil organic matter content (16.91 g kg^−1^), soil organic matter increased by 29.66% under BS100, 78.41% under BS100 + C, and 85.92% under BS75 + C. These results indicate that biochar addition substantially enhanced the capacity of biogas slurry to increase soil organic matter, with a magnitude far exceeding that achieved by biogas slurry alone. Furthermore, compared with the biochar-only treatment (CF + C), in which soil organic matter increased by 42.75% relative to the initial soil, the much greater increase observed under BS75 + C (85.92%) suggests that the combined application of biogas slurry and biochar promoted the formation of new soil organic matter, such as humus, rather than merely reflecting the direct carbon input from biochar. On the one hand, the porous structure of biochar increased soil porosity and enhanced N fertilizer use efficiency [33]. On the other hand, it provided physical protection for organic matter decomposition and microbial activity, thereby slowing the mineralization loss of organic matter and promoting the formation of stable humus [34]. These effects are consistent with the present finding that the BS75 + C treatment showed the highest soil organic matter content and together explain the advantage of this pattern in maintaining soil fertility and ensuring sustained nutrient supply, ultimately leading to increased yield.

### 4.2. Effects of Biogas Slurry–Biochar–Mineral Fertilizer Co-Application on the Net Ecosystem Carbon Balance and Its Components in the Greenhouse Farmland Ecosystem

The net ecosystem carbon balance (*N*_ECB_) is an accurate tool for evaluating short-term soil carbon balance in ecosystems [35]. In the present study, *N*_ECB_ estimation mainly included carbon input components, namely NPP-fixed carbon and fertilizer carbon input, and carbon output components, namely soil carbon emission and fruit carbon export. The present study found that NPP-fixed carbon was the dominant carbon input component in the farmland ecosystem. Among all treatments, BS75 + C showed the strongest carbon sequestration capacity and was significantly higher than the other treatments (*p* < 0.05), whereas fertilizer carbon input was highest in BS100. This may mainly be attributed to the nutrient supply system with both rapid and sustained release and enhanced storage capacity constructed through the synergistic interaction of biogas slurry, biochar, and mineral fertilizer. Specifically, an appropriate amount of mineral fertilizer can increase soil available N and total N contents, effectively promote crop root activity, and maintain soil microbial activity and abundance, thereby ensuring nutrient demand during the early growth stage of crops [36]. A high proportion of biogas slurry provides a sustained N source and active organic carbon, thereby enhancing the supply capacity of the soil carbon pool [37]. Biochar further prolongs nutrient availability and optimizes the rhizosphere microenvironment through nutrient adsorption and retention, soil structure improvement, and microbial activity regulation, thereby promoting the uptake of elements essential for chlorophyll synthesis, such as Mg, and comprehensively enhancing photosynthetic carbon assimilation and carbon allocation efficiency to different plant organs, especially roots [38,39]. By contrast, the biogas slurry-only treatment relied entirely on biogas slurry as the N source. Therefore, to achieve equal N input, the required amount of biogas slurry was the highest, resulting in the highest exogenous fertilizer carbon input. This objectively reflects the direct correlation between fertilizer carbon input and the proportion of organic fertilizer application. This result is consistent with the findings of Zhang et al. [40], who reported that organic fertilizer application can significantly increase *N*_ECB_ and enable agroecosystems to function as carbon sinks.

The results of the present study further showed that *N*_ECB_ was significantly affected by the regulation of carbon cycling processes under different fertilization patterns. In terms of carbon output, soil respiration was one of the major carbon emission pathways, while fruit carbon export also constituted an important component of system carbon output. Among all treatments, BS100 + C and BS50 + C exhibited relatively high soil respiration emissions throughout both growing seasons, with BS100 + C showing the highest values. Moreover, emissions were higher in the spring–summer season than in the autumn–winter season. This may be because the BS100 and BS50 treatments supplied large amounts of readily decomposable organic carbon, thereby markedly stimulating soil microbial activity [41], while the porous structure of biochar improved soil moisture content (Figure 2), further creating a favorable habitat for microorganisms. Together, these two factors synergistically enhanced the carbon mineralization rate [42], causing BS100 + C to exert the strongest priming effect on soil respiration. The stimulatory effect on CO_2_ emissions exceeded the inhibitory effect associated with carbon sequestration by biochar itself, especially under the higher temperature and moisture conditions of the spring–summer season. Unlike BS100 + C and BS50 + C, the BS75 + C treatment replaced mineral fertilizer at a more appropriate proportion, resulting in a more favorable carbon-to-nitrogen ratio, which enabled the carbon sequestration effect of the highly stable carbon fraction in biochar to be more fully expressed [6]. Therefore, although the high-rate biogas slurry combined with biochar treatment (BS75 + C) showed relatively high fruit carbon export, this treatment still exhibited the optimal *N*_ECB_ among all treatments, mainly because of its outstanding system carbon input capacity (Table 3). It is noteworthy that the highly stable carbon contained in the exogenously input biochar under the BS75 + C treatment not only directly increased the system carbon pool but was also scarcely involved in microbial decomposition, thereby effectively weakening the priming effect that might otherwise be induced by increased organic carbon input [43]. Therefore, the substitution of mineral fertilizer with high-rate biogas slurry combined with biochar achieved a balance between “high carbon input” and “controllable carbon output”, highlighting the unique advantage of the biogas slurry–biochar–mineral fertilizer integrated fertilization pattern in enhancing farmland carbon sink function.

### 4.3. Enhancement of Ecosystem Service Value in the Greenhouse Farmland Ecosystem by Biogas Slurry–Biochar–Mineral Fertilizer Co-Application

To quantify the ecological value of the farmland ecosystem, the present study referred to the Accounting Specifications for Gross Ecosystem Product and related literature, focusing on the valuation of its regulating services, mainly including soil conservation, nutrient retention, atmospheric regulation, and farmland moisture retention [15,44]. It is noteworthy that the BS75 + C treatment (75% biogas slurry combined with biochar) achieved the highest tomato yield in the present study, but did not exhibit the highest total ecosystem service value. In contrast, although the BS25 treatment (25% biogas slurry substitution for mineral fertilizer) produced a lower yield than BS75 + C, it achieved the highest total ecosystem service value because it provided the highest agricultural product supply value. Meanwhile, the 50% biogas slurry substitution treatment also ranked among the top treatments in the multi-objective comprehensive evaluation. The BS25 treatment achieved the highest total value mainly because the moderate nutrient supply pattern formed by partial organic substitution produced a favorable synergistic effect under the soil testing-based formula fertilization regime. Mineral fertilizer ensured the supply of readily available nutrients during critical growth stages [36], whereas the organic N, medium and trace elements, and physiologically active substances supplied by an appropriate amount of biogas slurry continued to play a role during the middle and late growth stages [45], thereby facilitating N transport and allocation to tomato fruits [46] and synergistically improving the agronomic efficiency of N fertilizer [47]. As a result, this treatment significantly reduced total input costs while maintaining stable yield and therefore achieved the optimal economic benefit, namely agricultural product supply value. In contrast, although BS75 + C achieved the highest yield through the combined effects of biochar and biogas slurry, the relatively high material cost of biochar together with the additional transportation and application costs associated with high-rate biogas slurry substitution weakened its economic performance. Given that agricultural product supply value dominated the total ecosystem service value (Figure S4), BS25 ultimately showed the highest total value.

By contrast, in terms of ecological regulating service value, the 75% biogas slurry combined with biochar treatment (BS75 + C) performed most prominently, particularly in organic matter accumulation and atmospheric regulation functions, including relatively high carbon sequestration and oxygen release capacities and relatively low greenhouse gas emissions. In addition, the combined weights of carbon sequestration value and oxygen release value ranked among the highest in the VIKOR comprehensive evaluation, contributing substantially to the superior overall performance of BS75 + C. This systematic advantage can mainly be attributed to the combined inputs of biochar and organic carbon from high-rate biogas slurry, which substantially increased both the recalcitrant and active carbon pools in soil and thereby enhanced soil carbon sequestration capacity [43,48]. At the same time, the nutrient supply system with both rapid and sustained release and enhanced storage capacity constructed under this treatment synchronously improved photosynthetic carbon assimilation and allocation efficiency, thereby contributing to higher net primary productivity and oxygen release [38,49]. In addition, biochar can effectively reduce greenhouse gas emissions such as N_2_O by improving soil aeration and adsorbing N [50,51], which is consistent with the findings of Iqbal et al. [12]. It should be noted that climate benefits depend not only on *N*_ECB_ but also on N_2_O emissions and carbon persistence (Figure S3). Under the BS75 + C treatment, N_2_O emissions were relatively low, and the carbon contained in biochar was highly stable, resulting in clear climate benefits. Overall, the carbon benefits brought about by biochar far exceeded its carbon costs, thus producing a positive net effect on climate change mitigation under the conditions of the present study [52].

### 4.4. Integrated Decision-Making Framework, Study Limitations, and Future Research Directions

In summary, the optimal treatment differed according to the evaluation objective. The results of the present study demonstrated significant differences among fertilization strategies in terms of economic benefits and carbon sink capacity, suggesting that management decisions should be tailored to specific objectives. If short-term economic benefit is prioritized, BS25 (25% biogas slurry substitution for mineral fertilizer) is recommended, as it achieved the highest total ecosystem service value, with BS50 (50% biogas slurry substitution for mineral fertilizer) ranking second. If the goal is the coordinated improvement of high and stable yield and ecological benefit, BS75 + C (75% biogas slurry combined with biochar) can be regarded as the optimal strategy, as it showed outstanding performance in net ecosystem carbon balance, soil organic matter accumulation, and atmospheric regulation capacity, and was identified as the optimal overall treatment by the VIKOR model based on AHP-CRITIC combined weighting. Nevertheless, several limitations should be acknowledged. First, the experiment was conducted in a single greenhouse using only one tomato cultivar, and therefore the generalizability of the conclusions requires further verification. Second, the biochar application rate was relatively high, and its economic feasibility and long-term effects warrant further evaluation. Third, certain carbon components, such as root exudates and changes in soil organic carbon stocks, were not included in the *N*_ECB_ calculation, which may introduce some uncertainty into the carbon budget assessment. In addition, tomato fruit quality attributes, including the sugar-to-acid ratio, vitamin C content, and nitrate concentration, were not evaluated, despite their direct relevance to market value and food safety. Future studies should therefore incorporate a multidimensional evaluation framework integrating quality, ecological, and economic indicators. Furthermore, although greenhouse gas sampling was intensified on days 1, 3, and 7 after the seedling establishment period, the subsequent 14-day sampling interval may still have been insufficient to fully capture short-term emission peaks following each biogas slurry application under practical production conditions characterized by frequent slurry inputs. Future studies should adopt higher sampling frequencies (e.g., at least once or twice per week) or automated continuous monitoring systems to improve the accuracy of greenhouse gas flux estimation. Overall, future research should include long-term, multi-location, and multi-cultivar experiments and integrate life cycle assessment approaches to comprehensively evaluate the sustainability of different fertilization strategies.

## 5. Conclusions

Different fertilization strategies significantly affected the net ecosystem carbon balance and ecosystem service value of the greenhouse farmland ecosystem, and the appropriate fertilization pattern varied with the production objective. When the objective was to maximize agricultural product supply value (economic benefit), 25% biogas slurry substitution for mineral fertilizer (BS25) performed best in both the spring–summer and autumn–winter seasons, reaching 641,606.83 and 629,987.37 CNY ha^−1^, respectively, while 50% biogas slurry substitution (BS50) ranked second. When the objective was to enhance carbon sink capacity and increase yield, 75% biogas slurry combined with biochar (BS75 + C) performed best, achieving the highest net ecosystem carbon balance while enhancing soil organic matter and atmospheric regulation. By contrast, neither biogas slurry alone nor mineral fertilizer combined with biochar produced superior overall effects. Furthermore, based on the VIKOR evaluation model with AHP-CRITIC combination weighting, BS75 + C was identified as the optimal option for enhancing carbon sink capacity and ecological benefits, whereas BS50 showed balanced performance across economic and ecological metrics. Overall, BS50 and BS75 + C can enhance ecological benefits and optimize fertilization schemes, supporting long-term agricultural sustainability and environmental protection.

## Figures and Tables

**Table 1:** Total fertilizer inputs and irrigation amounts under different experimental treatments.

Treatment	Total N (kg ha^−1^)			P_2_O_5_ (kg ha^−1^)			K_2_O (kg ha^−1^)			Irrigation Volume (m^3^ ha^−1^)	
	Biogas Slurry	Biochar	Chemical Nitrogen Fertilizer	Biogas Slurry	Biochar	Chemical Phosphate Fertilizer	Biogas Slurry	Biochar	Chemical Potassium Fertilizer	Spring–Summer	Autumn–Winter
CF	0.0	0.0	450.0	0.0	0.0	270.0	0.0	0.0	315.0	1818.9	1359.3
FR	0.0	0.0	380.0	0.0	0.0	180.0	0.0	0.0	500.0	1818.9	1359.3
BS25	95.0	0.0	285.0	12.3	0.0	167.7	27.5	0.0	472.5	1818.9	1359.3
BS50	190.0	0.0	190.0	24.6	0.0	155.4	55.0	0.0	445.0	1818.9	1359.3
BS75	285.0	0.0	95.0	36.9	0.0	143.1	82.5	0.0	417.5	1818.9	1359.3
BS100	380.0	0.0	0.0	49.2	0.0	130.8	110.0	0.0	390.0	1818.9	1359.3
CF + C	0.0	7.2	442.8	0.0	5.6	264.4	0.0	21.0	294.0	1818.9	1359.3
FR + C	0.0	7.2	372.8	0.0	5.6	174.4	0.0	21.0	479.0	1818.9	1359.3
BS25 + C	87.8	7.2	285.0	6.7	5.6	167.7	6.5	21.0	472.5	1818.9	1359.3
BS50 + C	182.8	7.2	190.0	19.0	5.6	155.4	34.0	21.0	445.0	1818.9	1359.3
BS75 + C	277.8	7.2	95.0	31.3	5.6	143.1	61.5	21.0	417.5	1818.9	1359.3
BS100 + C	372.8	7.2	0.0	43.6	5.6	130.8	89.0	21.0	390.0	1818.9	1359.3

**Table 2:** Effects of different biogas slurry–biochar–mineral fertilizer co-application treatments on soil organic matter content, biomass, yield, and cumulative soil carbon emissions. Data are presented as mean ± standard error (SE); different lowercase letters denote significant differences among treatments (*p* < 0.05).

Experimental Duration	Treatment	Soil Organic Matter Content (g kg^−1^)	Soil Organic Matter Content (t ha^−1^)	Biomass (t ha^−1^)	Yield (t ha^−1^)	Cumulative Soil CO_2_ Emissions (t ha^−1^)	Cumulative Soil CH_4_ Emissions (kg ha^−1^)
Spring– summer	CF	20.21 ± 0.48 j	50.10 ± 1.19 k	28.70 ± 1.32 b	119.36 ± 3.28 k	8.20 ± 0.38 g	−1.40 ± 0.14 ef
	FR	19.64 ± 0.36 k	47.31 ± 0.87 l	20.52 ± 0.85 j	102.58 ± 2.05 l	9.68 ± 0.42 ef	−0.33 ± 0.09 ab
	BS25	21.92 ± 0.52 i	50.16 ± 1.19 j	25.29 ± 1.12 d	142.60 ± 5.56 b	9.75 ± 0.40 ef	−1.09 ± 0.11 cd
	BS50	25.44 ± 1.03 g	60.40 ± 2.45 g	36.11 ± 1.54 a	130.91 ± 3.45 i	9.82 ± 0.57 e	−0.57 ± 0.08 b
	BS75	26.13 ± 1.22 f	60.34 ± 2.82 gh	22.42 ± 0.91 f	131.16 ± 2.17 h	10.08 ± 1.04 cd	−0.56 ± 0.07 b
	BS100	26.69 ± 1.19 e	60.97 ± 2.72 f	21.46 ± 0.86 i	125.97 ± 2.98 j	9.85 ± 0.85 de	−0.18 ± 0.02 a
	CF + C	24.28 ± 0.89 h	60.12 ± 2.20 i	36.19 ± 1.62 a	131.47 ± 4.07 g	10.16 ± 1.32 c	−2.68 ± 0.35 h
	FR + C	28.49 ± 1.36 d	66.31 ± 3.16 bc	21.72 ± 0.53 h	138.88 ± 3.49 d	9.50 ± 0.81 f	−1.30 ± 0.13 de
	BS25 + C	28.95 ± 1.40 c	65.22 ± 3.15 de	26.31 ± 0.74 c	135.09 ± 3.52 e	10.65 ± 1.14 b	−1.58 ± 0.24 f
	BS50 + C	29.17 ± 1.08 c	66.34 ± 2.46 b	24.36 ± 0.95 e	133.32 ± 2.48 f	11.46 ± 1.35 a	−2.24 ± 0.28 g
	BS75 + C	31.58 ± 1.64 a	72.26 ± 3.75 a	28.75 ± 1.29 b	152.39 ± 3.81 a	9.88 ± 0.93 de	−2.41 ± 0.32 g
	BS100 + C	30.31 ± 1.55 b	65.94 ± 3.37 d	22.13 ± 0.35 g	141.00 ± 4.94 c	11.53 ± 0.77 a	−1.02 ± 0.10 c
Autumn–winter	CF	19.98 ± 0.43 h	49.73 ± 1.07 h	28.08 ± 0.79 b	116.96 ± 2.47 k	7.45 ± 0.26 h	−1.45 ± 0.17 ef
	FR	19.41 ± 0.39 i	46.95 ± 0.94 j	19.90 ± 0.53 j	100.18 ± 1.38 l	8.72 ± 0.31 fg	−0.35 ± 0.12 ab
	BS25	21.86 ± 0.52 f	50.25 ± 1.20 g	24.66 ± 1.26 d	140.20 ± 5.62 b	8.51 ± 0.29 g	−1.11 ± 0.16 cd
	BS50	20.56 ± 0.47 g	49.01 ± 1.12 hi	35.49 ± 2.05 a	128.51 ± 3.02 i	8.59 ± 0.15 g	−0.59 ± 0.08 b
	BS75	17.28 ± 0.28 j	40.08 ± 0.65 k	21.79 ± 0.67 f	128.76 ± 3.14 h	9.01 ± 0.67 e	−0.60 ± 0.09 b
	BS100	17.16 ± 0.26 j	39.36 ± 0.60 l	20.83 ± 0.62 i	123.57 ± 2.79 j	9.31 ± 0.73 d	−0.21 ± 0.01 a
	CF + C	24.00 ± 0.85 e	59.65 ± 2.11 f	35.57 ± 1.85 a	129.37 ± 2.93 g	9.48 ± 0.82 cd	−2.75 ± 0.40 h
	FR + C	28.21 ± 1.42 d	65.93 ± 3.32 bc	21.10 ± 0.65 h	136.78 ± 3.54 d	8.75 ± 0.26 fg	−1.34 ± 0.15 de
	BS25 + C	28.66 ± 1.45 c	64.87 ± 3.28 e	25.68 ± 1.30 c	132.99 ± 1.95 e	9.67 ± 1.02 c	−1.62 ± 0.20 f
	BS50 + C	28.89 ± 1.53 c	65.98 ± 3.50 b	23.74 ± 1.14 e	131.22 ± 3.49 f	10.38 ± 0.96 b	−2.30 ± 0.21 g
	BS75 + C	31.30 ± 1.77 a	71.93 ± 4.06 a	28.12 ± 0.92 b	150.29 ± 6.18 a	8.88 ± 0.45 ef	−2.47 ± 0.34 g
	BS100 + C	30.03 ± 1.60 b	65.62 ± 3.50 cd	21.51 ± 0.73 g	138.90 ± 4.76 c	10.82 ± 0.97 a	−1.06 ± 0.11 c

**Table 3:** Effects of different biogas slurry–biochar–mineral fertilizer co-application treatments on the net ecosystem carbon balance and its composition in farmland ecosystems. Data are presented as mean ± standard error (SE); different lowercase letters denote significant differences among treatments (*p* < 0.05).

Experimental Duration	Treatment	NPP-Fixed Carbon (t ha^−1^)	Fertilizer Carbon Input (t ha^−1^)	Fruit Carbon Export (t ha^−1^)	Soil Carbon Output (t ha^−1^)	Net Ecosystem Carbon Balance (t ha^−1^)
Spring–summer	CF	12.08 ± 0.66 g	0.1957	1.88 ± 0.15 bc	8.16 ± 0.52 g	2.24 ± 0.16 d
	FR	11.12 ± 0.54 h	0.1652	1.59 ± 0.13 ef	9.67 ± 0.61 ef	0.03 ± 0.01 i
	BS25	11.03 ± 0.51 h	0.2085	0.96 ± 0.08 g	9.72 ± 0.47 ef	0.56 ± 0.02 g
	BS50	17.05 ± 0.73 c	0.2517	1.63 ± 0.14 def	9.81 ± 0.63 de	5.87 ± 0.20 b
	BS75	12.70 ± 0.62 g	0.2950	1.82 ± 0.07 bcd	10.06 ± 1.05 cd	1.12 ± 0.09 e
	BS100	11.02 ± 0.48 h	0.3382	0.75 ± 0.06 g	9.85 ± 0.74 cde	0.77 ± 0.06 f
	CF + C	17.58 ± 1.43 b	0.1960	1.49 ± 0.10 f	10.08 ± 1.14 c	6.21 ± 0.19 a
	FR + C	11.00 ± 0.27 h	0.1656	1.56 ± 0.12 ef	9.46 ± 0.38 f	0.13 ± 0.01 hi
	BS25 + C	14.93 ± 0.71 d	0.2055	1.58 ± 0.08 ef	10.61 ± 1.25 b	2.96 ± 0.13 c
	BS50 + C	13.22 ± 0.64 f	0.2488	1.99 ± 0.15 b	11.39 ± 0.96 a	0.08 ± 0.02 i
	BS75 + C	18.09 ± 1.45 a	0.2920	2.26 ± 0.23 a	9.82 ± 0.72 de	6.30 ± 0.24 a
	BS100 + C	13.54 ± 0.38 e	0.3353	1.99 ± 0.20 b	11.50 ± 0.80 a	0.38 ± 0.03 gh
Autumn–winter	CF	11.20 ± 0.47 g	0.1957	1.84 ± 0.18 bc	7.41 ± 0.28 h	2.15 ± 0.14 d
	FR	10.16 ± 0.39 h	0.1652	1.55 ± 0.12 de	8.71 ± 0.37 fg	0.07 ± 0.01 h
	BS25	9.71 ± 0.11 i	0.2085	0.93 ± 0.02 f	8.48 ± 0.32 g	0.50 ± 0.05 fg
	BS50	15.99 ± 0.58 b	0.2517	1.60 ± 0.14 cde	8.57 ± 0.46 fg	6.07 ± 1.79 b
	BS75	11.88 ± 0.49 f	0.2950	1.78 ± 0.16 bcd	9.00 ± 0.65 e	1.39 ± 0.12 e
	BS100	10.25 ± 0.22 h	0.3382	0.72 ± 0.05 f	9.31 ± 0.72 d	0.56 ± 0.07 f
	CF + C	16.93 ± 1.04 a	0.1960	1.46 ± 0.10 e	9.40 ± 1.08 cd	6.26 ± 2.03 ab
	FR + C	10.23 ± 0.19 h	0.1656	1.53 ± 0.11 de	8.71 ± 0.79 fg	0.15 ± 0.01 h
	BS25 + C	14.01 ± 0.55 c	0.2055	1.55 ± 0.19 de	9.63 ± 1.22 c	3.04 ± 0.16 c
	BS50 + C	12.34 ± 0.50 e	0.2488	1.96 ± 0.25 b	10.32 ± 0.95 b	0.32 ± 0.02 fgh
	BS75 + C	17.08 ± 0.96 a	0.2920	2.22 ± 0.30 a	8.82 ± 0.34 ef	6.34 ± 1.92 a
	BS100 + C	12.69 ± 0.43 d	0.3353	1.96 ± 0.21 b	10.79 ± 0.82 a	0.28 ± 0.02 gh

**Table 4:** Effects of different biogas slurry–biochar–mineral fertilizer co-application treatments on agricultural product supply value (economic benefit) and its composition. Different lowercase letters denote significant differences among treatments (*p* < 0.05).

Experimental Duration	Treatment	Mineral Fertilizer Cost (CNY ha^−1^)	Biogas Slurry Cost (CNY ha^−1^)	Biochar Cost (CNY ha^−1^)	Seedling Cost (CNY ha^−1^)	Irrigation Cost (CNY ha^−1^)	Total Input Cost (CNY ha^−1^)	Yield Revenue (CNY ha^−1^)	Economic Benefit (CNY ha^−1^)
Spring–summer	CF	6485.58	0.00	0.00	41,142.00	3183.08	50,810.66	580,109.04	529,298.39 e
	FR	7912.16	0.00	0.00	41,142.00	3183.08	52,237.24	498,541.23	446,304.00 g
	BS25	7089.70	7762.97	0.00	41,142.00	3009.17	59,003.85	693,045.72	634,041.87 a
	BS50	6267.25	15,525.94	0.00	41,142.00	2835.27	65,770.46	636,205.59	570,435.13 b
	BS75	5444.79	23,288.91	0.00	41,142.00	2661.37	72,537.08	637,437.60	564,900.52 c
	BS100	4622.34	31,051.88	0.00	41,142.00	2487.47	79,303.69	612,214.20	532,910.51 d
	CF + C	6203.53	0.00	180,000.00	41,142.00	3183.08	230,528.61	638,924.76	408,396.15 k
	FR + C	7630.11	0.00	180,000.00	41,142.00	3183.08	231,955.19	674,951.94	442,996.75 h
	BS25 + C	7089.70	7135.67	180,000.00	41,142.00	3009.17	238,376.54	656,522.82	418,146.28 j
	BS50 + C	6267.25	14,898.64	180,000.00	41,142.00	2835.27	245,143.16	647,957.07	402,813.91 l
	BS75 + C	5444.79	22,661.61	180,000.00	41,142.00	2661.37	251,909.77	740,620.26	488,710.49 f
	BS100 + C	4622.34	30,424.58	180,000.00	41,142.00	2487.47	258,676.39	685,260.00	426,583.61 i
Autumn–winter	CF	6485.58	0.00	0.00	41,142.00	2378.78	50,006.36	568,445.04	518,438.69 e
	FR	7912.16	0.00	0.00	41,142.00	2378.78	51,432.94	486,877.23	435,444.3 g
	BS25	7089.70	7762.97	0.00	41,142.00	2204.87	58,199.55	681,381.72	623,182.17 a
	BS50	6267.25	15,525.94	0.00	41,142.00	2030.97	64,966.16	624,541.59	559,575.43 b
	BS75	5444.79	23,288.91	0.00	41,142.00	1857.07	71,732.78	625,773.60	554,040.82 c
	BS100	4622.34	31,051.88	0.00	41,142.00	1683.17	78,499.39	600,550.20	522,050.81 d
	CF + C	6203.53	0.00	180,000.00	41,142.00	2378.78	229,724.31	628,718.76	398,994.45 k
	FR + C	7630.11	0.00	180,000.00	41,142.00	2378.78	231,150.89	664,745.94	433,595.05 h
	BS25 + C	7089.70	7135.67	180,000.00	41,142.00	2204.87	237,572.24	646,316.82	408,744.58 j
	BS50 + C	6267.25	14,898.64	180,000.00	41,142.00	2030.97	244,338.86	637,751.07	393,412.21 l
	BS75 + C	5444.79	22,661.61	180,000.00	41,142.00	1857.07	251,105.47	730,414.26	479,308.79 f
	BS100 + C	4622.34	30,424.58	180,000.00	41,142.00	1683.17	257,872.09	675,054.00	417,181.91 i

**Table 5:** Effects of different biogas slurry–biochar–mineral fertilizer co-application treatments on ecosystem service value and its composition in farmland ecosystems. Different lowercase letters denote significant differences among treatments (*p* < 0.05).

Experimental Duration	Treatment	Agricultural Product Supply Value (CNY ha^−1^)	Organic Matter Accumulation Value (CNY ha^−1^)	Atmospheric Regulation Value				Ecosystem Service Value (CNY ha^−1^)
				Carbon Sequestration Value (CNY ha^−1^)	Oxygen Release Value (CNY ha^−1^)	Greenhouse Gas Emission Value (CNY ha^−1^)	Total (CNY ha^−1^)	
Spring- summer	CF	529,298.39 e	1285.08 k	3383.21 h	5462.64 h	−1800.05 k	7045.79 h	537,629.26 e
	FR	446,304.00 g	1213.44 l	3115.19 i	5029.89 i	−1816.70 h	6328.38 i	453,845.82 g
	BS25	634,041.87 a	1286.59 j	3090.2 j	4989.55 j	−1801.39 j	6278.36 k	641,606.83 a
	BS50	570,435.13 b	1549.27 g	4776.47 c	7712.25 c	−1831.72 g	10,657.00 b	582,641.40 b
	BS75	564,900.52 c	1547.69 h	3557.65 g	5744.30 g	−1802.03 i	7499.92 e	573,948.13 c
	BS100	532,910.51 d	1564.00 f	3086.32 k	4983.28 k	−1761.35 l	6308.26 j	540,782.77 d
	CF + C	408,396.15 k	1541.99 i	4924.18 b	7950.75 b	−2538.99 c	10,335.94 c	420,274.08 k
	FR + C	442,996.75 h	1700.75 c	3080.22 l	4973.42 l	−2284.28 e	5769.36 l	450,466.86 h
	BS25 + C	418,146.28 j	1672.97 e	4182.61 d	6753.38 d	−2448.11 d	8487.89 d	428,307.13 j
	BS50 + C	402,813.91 l	1701.69 b	3701.18 f	5976.05 f	−2588.31 a	7088.91 g	411,604.51 l
	BS75 + C	488,710.49 f	1853.57 a	5066.15 a	8179.98 a	−2259.10 f	10,987.03 a	501,551.08 f
	BS100 + C	426,583.61 i	1691.39 d	3792.02 e	6122.72 e	−2569.34 b	7345.40 f	435,620.41 i
Autumn- winter	CF	518,438.69 e	1275.68 h	3136.02 h	5063.52 h	−1599.79 k	6599.75 h	526,314.12 e
	FR	435,444.3 g	1204.31 j	2845.21 k	4593.96 k	−1640.33 h	5798.84 j	442,447.44 g
	BS25	623,182.17 a	1288.91 g	2719.30 l	4390.68 l	−1593.69 l	5516.29 k	629,987.37 a
	BS50	559,575.43 b	1257.19 i	4478.27 c	7230.76 c	−1610.92 j	10,098.10 b	570,930.72 b
	BS75	554,040.82 c	1027.93 k	3326.64 g	5371.31 g	−1615.10 i	7082.86 e	562,151.61 c
	BS100	522,050.81 d	1009.57 l	2871.07 i	4635.72 i	−1673.88 g	5832.91 i	528,893.29 d
	CF + C	398,994.45 k	1530.06 f	4740.77 b	7654.60 b	−2380.85 c	10,014.53 c	410,539.04 k
	FR + C	433,595.05 h	1690.98 c	2864.6 j	4625.28 j	−2144.78 e	5345.1 l	440,631.13 h
	BS25 + C	408,744.58 j	1663.86 e	3922.46 d	6333.33 d	−2273.30 d	7982.49 d	418,390.93 j
	BS50 + C	393,412.21 l	1692.49 b	3456.56 f	5581.08 f	−2395.85 b	6641.79 g	401,746.49 l
	BS75 + C	479,308.79 f	1844.88 a	4783.99 a	7724.39 a	−2084.96 f	10,423.42 a	491,577.09 f
	BS100 + C	417,181.91 i	1683.21 d	3554.72 e	5739.58 e	−2447.83 a	6846.47 f	425,711.59 i

**Table 6:** Ranking of agricultural product supply value, carbon sequestration value, oxygen release value, greenhouse gas emission value, farmland moisture retention value, organic matter accumulation value, and the comprehensive compromise index (*Q_i_*) based on the VIKOR evaluation model. *R*^+^ and *R*^−^ represent the positive ideal solution and negative ideal solution, respectively.

Treatment	Agricultural Product Supply Value (CNY ha^−1^)	Carbon Sequestration Value (CNY ha^−1^)	Oxygen Release Value (CNY ha^−1^)	Greenhouse Gas Emission Value (CNY ha^−1^)	Farmland Moisture Retention Value (CNY ha^−1^)	Organic Matter Accumulation Value (CNY ha^−1^)	*S_i_*	*R_i_*	*Q_i_*	Ranking
CF	0.3092	0.2506	0.2506	−0.2372	0.2481	0.2455	0.7262	0.2606	0.7631	8
FR	0.2602	0.2291	0.2291	−0.2412	0.2604	0.2318	0.8869	0.3043	0.9728	12
BS25	0.3710	0.2233	0.2233	−0.2369	0.2718	0.2469	0.7303	0.3161	0.8939	9
BS50	0.3334	0.3557	0.3557	−0.2402	0.2776	0.2690	0.2591	0.0991	0.0744	2
BS75	0.3302	0.2646	0.2646	−0.2385	0.2827	0.2469	0.6278	0.2320	0.6306	7
BS100	0.3113	0.2290	0.2290	−0.2397	0.2790	0.2467	0.7900	0.3045	0.9077	10
CF + C	0.2382	0.3715	0.3715	−0.3433	0.2816	0.2945	0.3488	0.1841	0.3311	3
FR + C	0.2587	0.2285	0.2285	−0.3091	0.3034	0.3251	0.7953	0.3055	0.9135	11
BS25 + C	0.2440	0.3115	0.3115	−0.3295	0.3081	0.3199	0.5178	0.1761	0.4273	4
BS50 + C	0.2349	0.2751	0.2751	−0.3478	0.2959	0.3254	0.6746	0.2106	0.6131	6
BS75 + C	0.2856	0.3786	0.3786	−0.3031	0.3232	0.3545	0.1493	0.1184	0.0444	1
BS100 + C	0.2490	0.2824	0.2824	−0.3501	0.3221	0.3235	0.6193	0.1958	0.5415	5
*R^+^*	0.3710	0.3786	0.3786	−0.2369	0.3232	0.3545				
*R* ^−^	0.2349	0.2233	0.2233	−0.3501	0.2481	0.2318				
Combined weight	0.1887	0.3161	0.2663	0.0528	0.0338	0.1423				
Subjective weight	0.2201	0.2193	0.1847	0.0491	0.1451	0.1817				
Objective weight	0.1469	0.2472	0.2472	0.1846	0.0400	0.1342				

## Data Availability

The original contributions presented in this study are included in the article/Supplementary Materials. Further inquiries can be directed to the corresponding authors.

## Supplementary Materials

The following supporting information can be downloaded at https://www.mdpi.com/article/10.3390/plants15132087/s1, Table S1. Soil physicochemical properties of the 0–20 cm soil layer before tomato transplantation. Table S2. Physicochemical properties of the tested biogas slurry and biochar. Figure S1. Chord diagram of the flow relationships among the components of the net ecosystem carbon balance of the farmland ecosystem under different treatments. Each sector represents a different component of the net ecosystem carbon balance of the farmland ecosystem, and each chord represents the flow relationship and its relative strength between components. The width of each chord is proportional to the corresponding carbon flux. Figure S2. Sankey diagram of the contribution distribution of each component of agricultural product supply value in different treatment groups. The left side shows the source factors, and the right side shows the treatment groups. The width of each flow is proportional to the relative contribution of the corresponding source factor to the target treatment group. Figure S3. Effects of different treatments on soil CO_2_, N_2_O, and CH_4_ emissions. Red arrows indicate the fertilization dates. Different lowercase letters indicate significant differences among treatments. Figure S4. Sankey diagram of the contribution distribution of each component of ecosystem service value in different treatment groups. The left side shows the source factors, and the right side shows the treatment groups. The width of each flow is proportional to the relative contribution of the corresponding source factor to the target treatment group.
