# Supplementary material for: Effects of Biogas Slurry, Biochar, and Mineral Fertilizer Co-Application on Net Ecosystem Carbon Balance and Ecosystem Service Value in Greenhouse Farmland
Source: Plants (Basel). 2026 Jul 4;15(13):2087. doi: 10.3390/plants15132087 (PMC13363808; doi:10.3390/plants15132087)
Supplement: Supplementary file 1 [file plants-15-02087-s001.zip › plants-4359135-supplementary.pdf]

Table S1. Soil physicochemical properties of the 0–20 cm soil layer before tomato transplantation.

Soil property	Value
Bulk density (g cm^{-3})	1.28
Soil moisture content (%)	19.93
pH value	7.61
Organic carbon content (g kg^{-1})	9.81
Total nitrogen content (g kg^{-1})	0.85
Total phosphorus content (g kg^{-1})	2.04
Alkaline hydrolyzable nitrogen content (mg kg^{-1})	58.74
Available potassium content (mg kg^{-1})	130.36
Microbial biomass carbon (mg kg^{-1})	190.01

Table S2. Physicochemical properties of the tested biogas slurry and biochar.

Returned field samples	Test items	Content
Biogas slurry	Total solids (TS) content (%)	2.00
	Organic carbon content (g L^{-1})	0.99
	pH value	7.78
	Alkali-hydrolyzable nitrogen content (g L^{-1})	0.89
	Total nitrogen content (g L^{-1})	0.96
	Total phosphorus content (g L^{-1})	0.05
	Total potassium content (g L^{-1})	0.23
	Volatile fatty acid (VFA) concentration (mg L^{-1})	1913.53
	NH_4^+-N content (mg L^{-1})	146.00
	NO_3^--N content (mg L^{-1})	31.14
Biochar	Carbon content (%)	48.19
	Nitrogen content (%)	0.80
	C:N ratio	60.24
	Trace elements (Zn, B, Mo, etc.) (%)	2.00
	Moisture content (%)	28.36
	pH value	9.04
	Organic matter content (g kg^{-1})	925.74
	Alkali-hydrolyzable nitrogen content (g kg^{-1})	0.16
	Available phosphorus content (g kg^{-1})	0.05
	Available potassium content (g kg^{-1})	0.38
	Specific surface area (m^2 g^{-1})	52.38
	Total porosity (%)	63.14
	Aeration porosity (%)	11.36
	Water-holding porosity (%)	51.78
	Volatile matter (%)	31.63

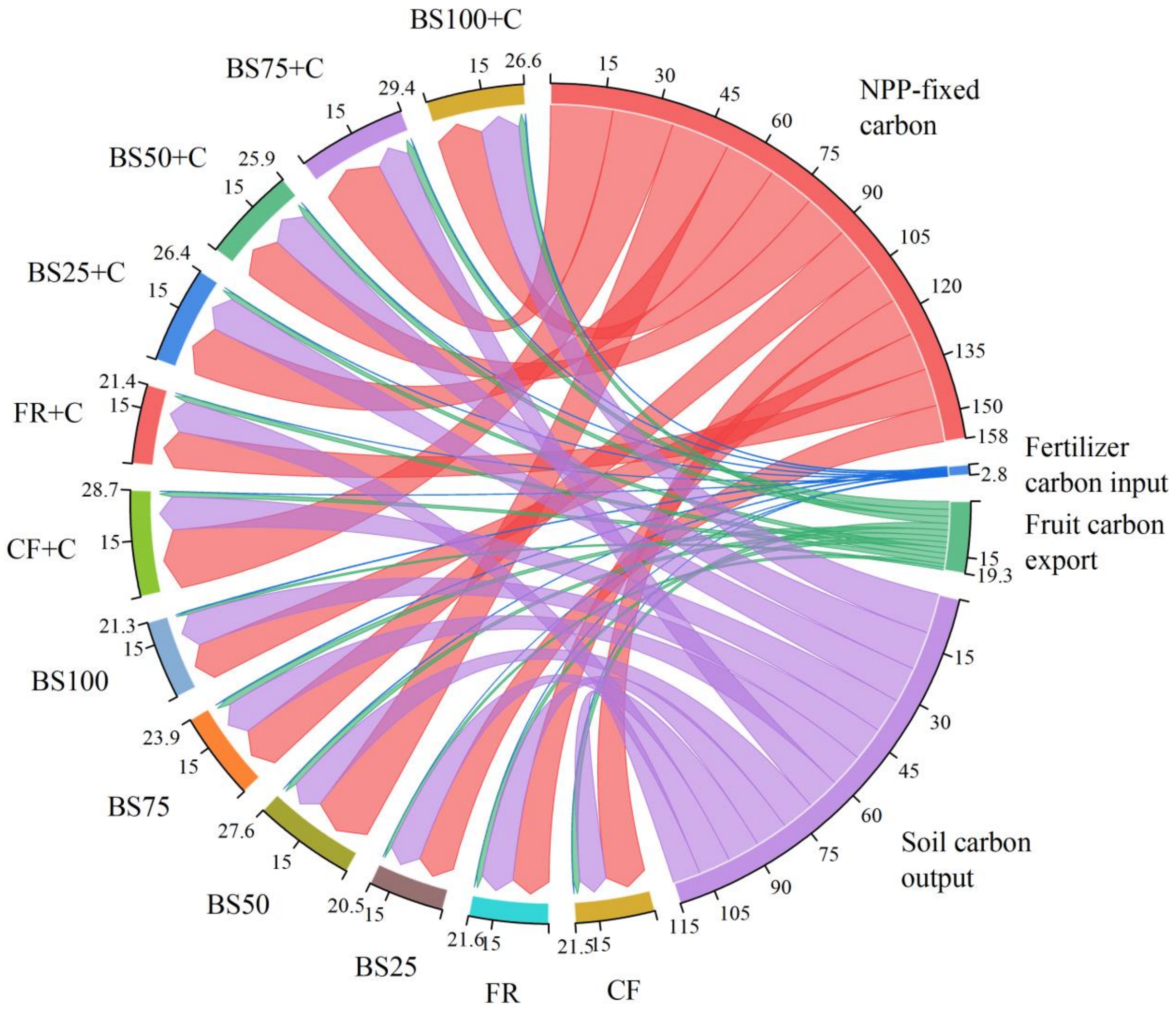

Figure S1. Chord diagram of the flow relationships among the components of the net ecosystem carbon balance of the farmland ecosystem under different treatments. Each sector represents a different component of the net ecosystem carbon balance of the farmland ecosystem, and each chord represents the flow relationship and its relative strength between components. The width of each chord is proportional to the corresponding carbon flux.

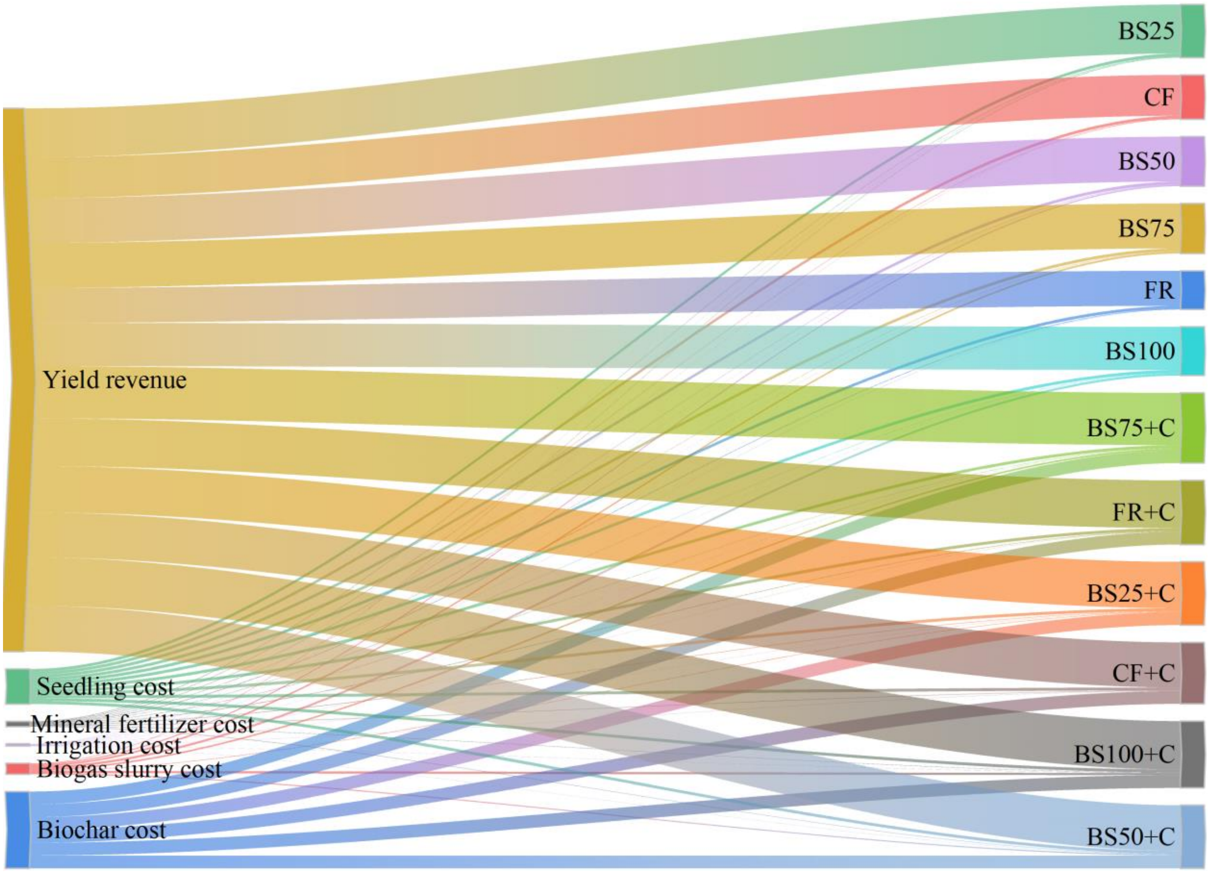

Figure S2. Sankey diagram of the contribution distribution of each component of agricultural product supply value in different treatment groups. The left side shows the source factors, and the right side shows the treatment groups. The width of each flow is proportional to the relative contribution of the corresponding source factor to the target treatment group.

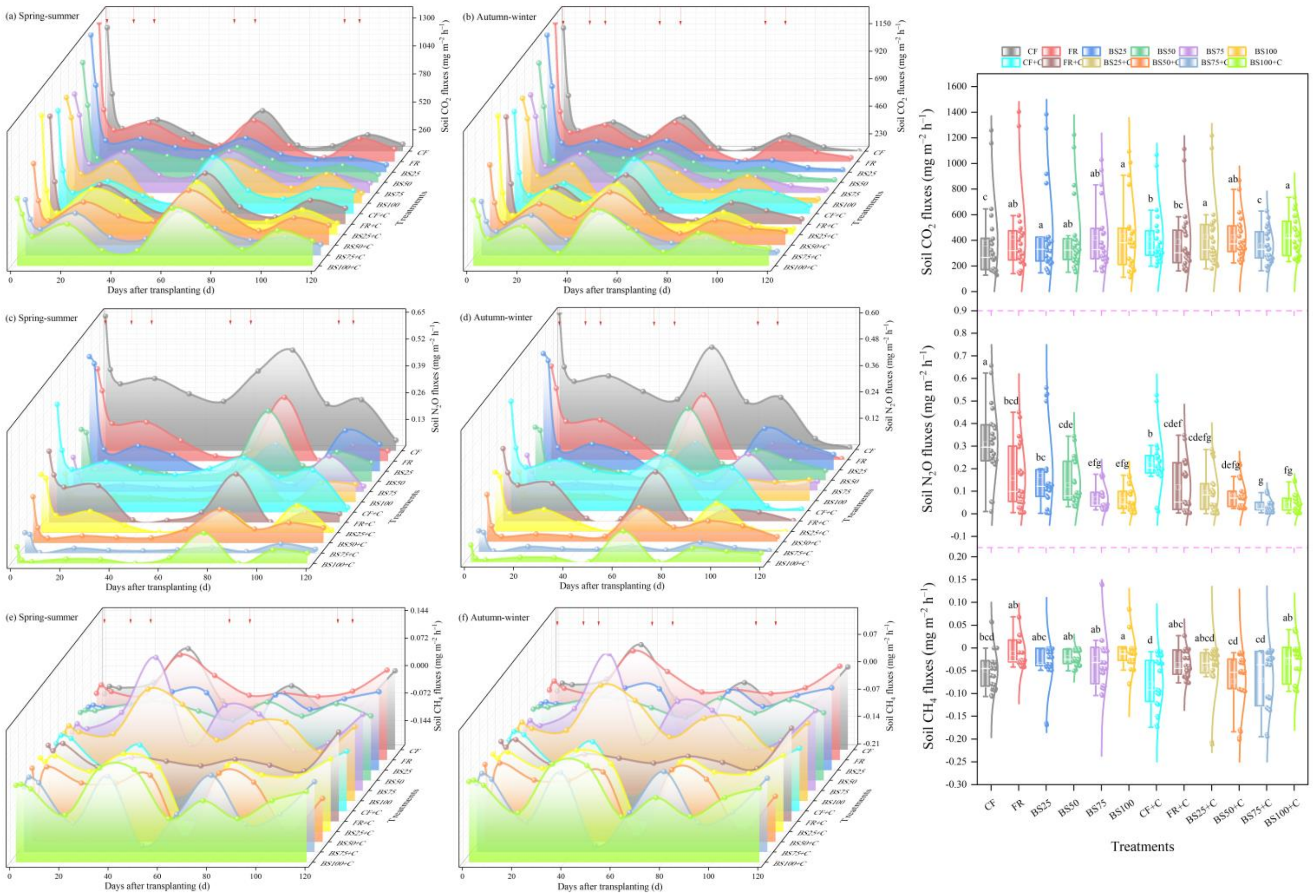

Figure S3. Effects of different treatments on soil CO_2, N_2O, and CH_4 emissions. Red arrows indicate the fertilization dates. Different lowercase letters indicate significant differences among treatments.

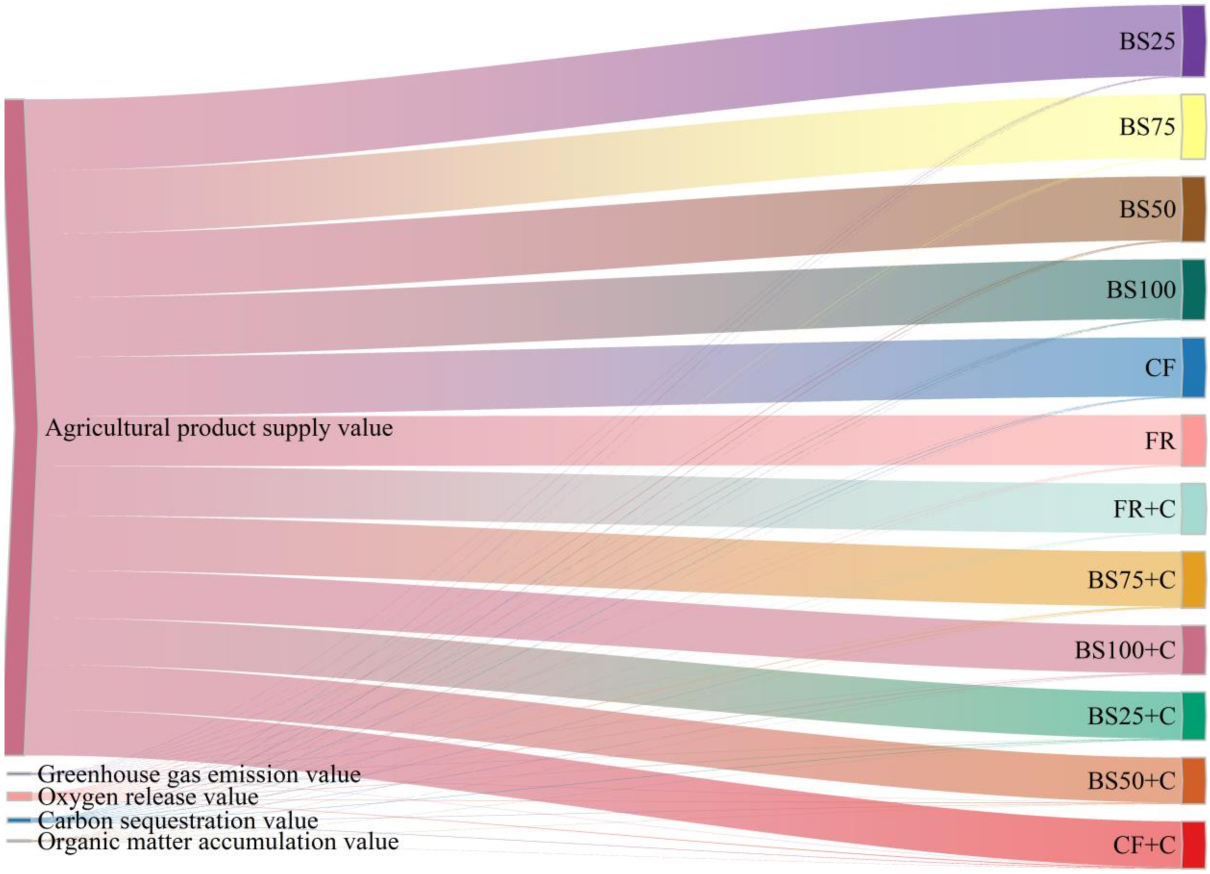

Figure S4. Sankey diagram of the contribution distribution of each component of ecosystem service value in different treatment groups. The left side shows the source factors, and the right side shows the treatment groups. The width of each flow is proportional to the relative contribution of the corresponding source factor to the target treatment group.
